# The fibronectin-targeting PEG-FUD imaging probe shows enhanced uptake during fibrogenesis in experimental lung fibrosis

**DOI:** 10.1186/s12931-025-03107-x

**Published:** 2025-01-22

**Authors:** Thomas J. Harr, Nikesh Gupta, Babita Rahar, Kristen Stott, Yadira Medina-Guevara, Metti K. Gari, Angie T. Oler, Ivy Sohee McDermott, Hye Jin Lee, Morteza Rasoulianboroujeni, Ashley M. Weichmann, Amir Forati, Kelsey Holbert, Trevor S. Langel, Kade W. Coulter, Brian M. Burkel, Bianca R. Tomasini-Johansson, Suzanne M. Ponik, Jonathan W. Engle, Reinier Hernandez, Glen S. Kwon, Nathan Sandbo, Ksenija Bernau

**Affiliations:** 1 Division of Allergy, Pulmonary and Critical Care Medicine, Department of Medicine, School of Medicine and Public Health, University of Wisconsin-Madison, 600 Highland Avenue, Madison, WI 53792 USA; 2 Pharmaceutical Sciences Division, School of Pharmacy, University of Wisconsin-Madison, 777 Highland Avenue, Madison, WI 53705 USA; 3 Department of Medical Physics, School of Medicine and Public Health, University of Wisconsin-Madison, 1111 Highland Avenue, Madison, WI 53705 USA; 4 Department of Cell and Regenerative Biology, School of Medicine and Public Health, University of Wisconsin-Madison, 1111 Highland Avenue, Madison, WI 53705 USA; 5 University of Wisconsin Carbone Cancer Center, University of Wisconsin-Madison, 1111 Highland Avenue, Madison, WI USA; 6 Department of Medicine, School of Medicine and Public Health, University of Wisconsin- Madison, 600 Highland Avenue, Madison, WI 53792 USA; 7 College of Pharmacy, Chungbuk National University, Cheongju, 28160 Republic of Korea

**Keywords:** Recombinant peptide, Pulmonary fibrosis, Imaging, Non-invasive biomarker, Fibronectin

## Abstract

Progressive forms of interstitial lung diseases, including idiopathic pulmonary fibrosis (IPF), are deadly disorders lacking non-invasive biomarkers for assessment of early disease activity, which presents a major obstacle in disease management. Excessive extracellular matrix (ECM) deposition is a hallmark of these disorders, with fibronectin being an abundant ECM glycoprotein that is highly upregulated in early fibrosis and serves as a scaffold for the deposition of other matrix proteins. Due to its role in active fibrosis, we are targeting fibronectin as a biomarker of early lung fibrosis disease activity via the PEGylated fibronectin-binding polypeptide (PEG-FUD). In this work, we demonstrate the binding of PEG-FUD to the fibrotic lung throughout the course of bleomycin-induced murine model of pulmonary fibrosis. We first analyzed the binding of radiolabeled PEG-FUD following direct incubation to precision cut lung slices from mice at different stages of experimental lung fibrosis. Then, we administered fluorescently labeled PEG-FUD subcutaneously to mice over the course of bleomycin-induced pulmonary fibrosis and assessed peptide uptake 24 h later through ex vivo tissue imaging. Using both methods, we found that peptide targeting to the fibrotic lung is increased during the fibrogenic phase of the single dose bleomycin lung fibrosis model (days 7 and 14 post-bleomycin). At these timepoints we found a correlative relationship between peptide uptake and fibrotic burden. These data suggest that PEG-FUD targets fibronectin associated with active fibrogenesis in this model, making it a promising candidate for a clinically translatable molecular imaging probe to non-invasively determine pulmonary fibrosis disease activity, enabling accelerated therapeutic decision-making.

**Supplementary Information:**

The online version contains supplementary material available at 10.1186/s12931-025-03107-x.

## Introduction

Idiopathic pulmonary fibrosis (IPF) is a progressive and irreversible form of interstitial lung disease (ILD) with median survival duration of 2 to 5 years after diagnosis [1, 2]. In addition to IPF, other ILDs, including hypersensitivity pneumonitis, nonspecific interstitial pneumonia and connective-tissue related ILDs, may develop into progressive disease described by worsening respiratory symptoms, decline in lung function, increasing fibrosis on high resolution computed tomography (HRCT), and premature mortality [3]. The absence of reliable non-invasive biomarkers for early detection of active disease progression is a major hurdle in the care of patients with these diseases. Current methods for assessing disease progression, including HRCT and pulmonary function tests, can only provide retrospective monitoring of disease activity by comparing tests from months prior [4, 5]. Development of biomarkers with high fidelity to disease activity is a major need in the field, with active research into characteristic transcriptional, proteomic, and metabolic signatures that predict disease progression [6–8]. An ideal biomarker for progressive pulmonary fibrosis would provide unique, real-time information about that disease state (activity level), offering additive information to currently utilized methods that are able to ascertain overall fibrotic burden.

In addition to traditional biospecimen-based biomarkers, imaging biomarkers are a potentially advantageous approach to non-invasively probe disease processes, such as aberrant disease mechanisms, including enhanced glucose metabolisms [9–11], cellular activity [12–16], and extracellular matrix (ECM) deposition [17–20]. Since ECM deposition is a hallmark in the development of fibrosing disorders, molecular imaging probes that target mechanisms of ECM deposition hold promise for detection of disease activity. In particular, the ECM glycoprotein fibronectin (FN) plays a pivotal role in the development of pulmonary fibroses, where it serves as a key regulator in the deposition of collagen and other ECM components [21–24]. Its high and differential upregulation in fibrotic tissues, especially in the ECM producing fibroblastic foci in IPF, and the association of elevated FN levels with pulmonary fibrosis disease progression, mark it as a potentially useful target to monitor clinical disease activity [6, 23–25].

**Fig. 1:**
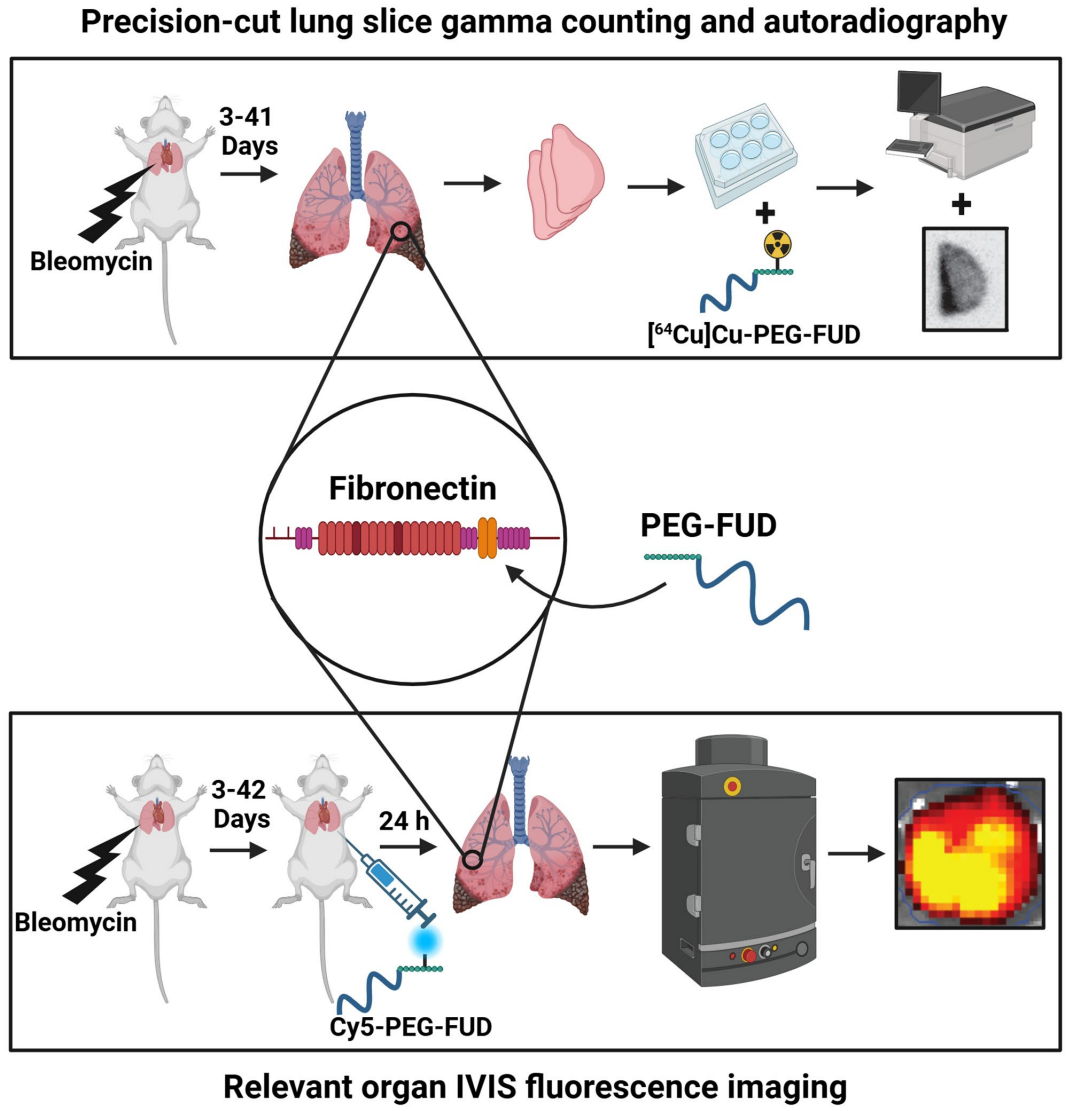
Study experimental design. Bleomycin was intratracheally delivered to mouse lungs to induce fibrosis, characterized by increased fibronectin expression. PEGylated FUD (PEG-FUD) peptide targets the N-terminal 70 kDa region of fibronectin and has been examined as an imaging probe for pulmonary fibrosis. To understand the temporal uptake of PEG-FUD in the bleomycin-induced model of pulmonary fibrosis, two approaches were utilized at various time points throughout this model. Top: precision-cut lung slices from fibrotic mouse lungs were incubated with radiolabeled peptide ([^64^Cu]Cu-PEG-FUD) and radioactivity was quantified via gamma counting and autoradiography. Bottom: Fluorescently labeled peptide (Cy5-PEG-FUD) was delivered to mice subcutaneously, followed by relevant organ ex vivo IVIS fluorescence imaging and signal quantification. Figure was created in BioRender.com

The functional upstream domain (FUD, also known as pUR4), is a 6-kD peptide derived from *Streptococcus pyogenes* F1 adhesin that has high binding affinity to the N-terminus of FN and shows promise as a molecular probe to target FN in the context of pulmonary fibrosis [19, 26, 27]. By modifying FUD with 20-kD polyethylene glycol (PEG), while preserving its nanomolar affinity for the N-terminal 70-kD domain of FN, we have improved its stability and circulation time, thereby enhancing its ability to target FN-rich areas in fibrotic tissues in vivo [28–30]. Our previous work demonstrated the capacity of PEG-FUD to serve as a molecular imaging probe for pulmonary fibrosis [19]. However, the dynamics of lung uptake of PEG-FUD during different phases of lung fibrosis development and resolution are unknown. Therefore, this holds implications for the utility of the probe in eventual clinical practice. Here, we characterize the differential targeting of our peptide during the temporal evolution of the bleomycin-induced murine model of pulmonary fibrosis by determining both direct binding of radiolabeled PEG-FUD on precision cut lung slices (PCLS) from fibrotic lungs and localization of fluorescently-labeled peptide in ex vivo lung tissues 24 h after in vivo delivery (Fig. 1).

We show that PEG-FUD probe uptake is increased during the fibrogenic phase of bleomycin-induced lung fibrosis and that the level of peptide targeting correlates with overall fibrosis during these stages. These findings provide further support for the use of PEG-FUD as a probe for early disease activity in lung fibrosis, additionally validating its potential translation to clinical use, where it could offer pulmonary care teams more detailed and accurate insight into patients’ disease status and the need for therapeutic intervention. Portions of this data have previously been presented in abstract form [31–33].

## Methods

### Study design

**Fig. 2:**
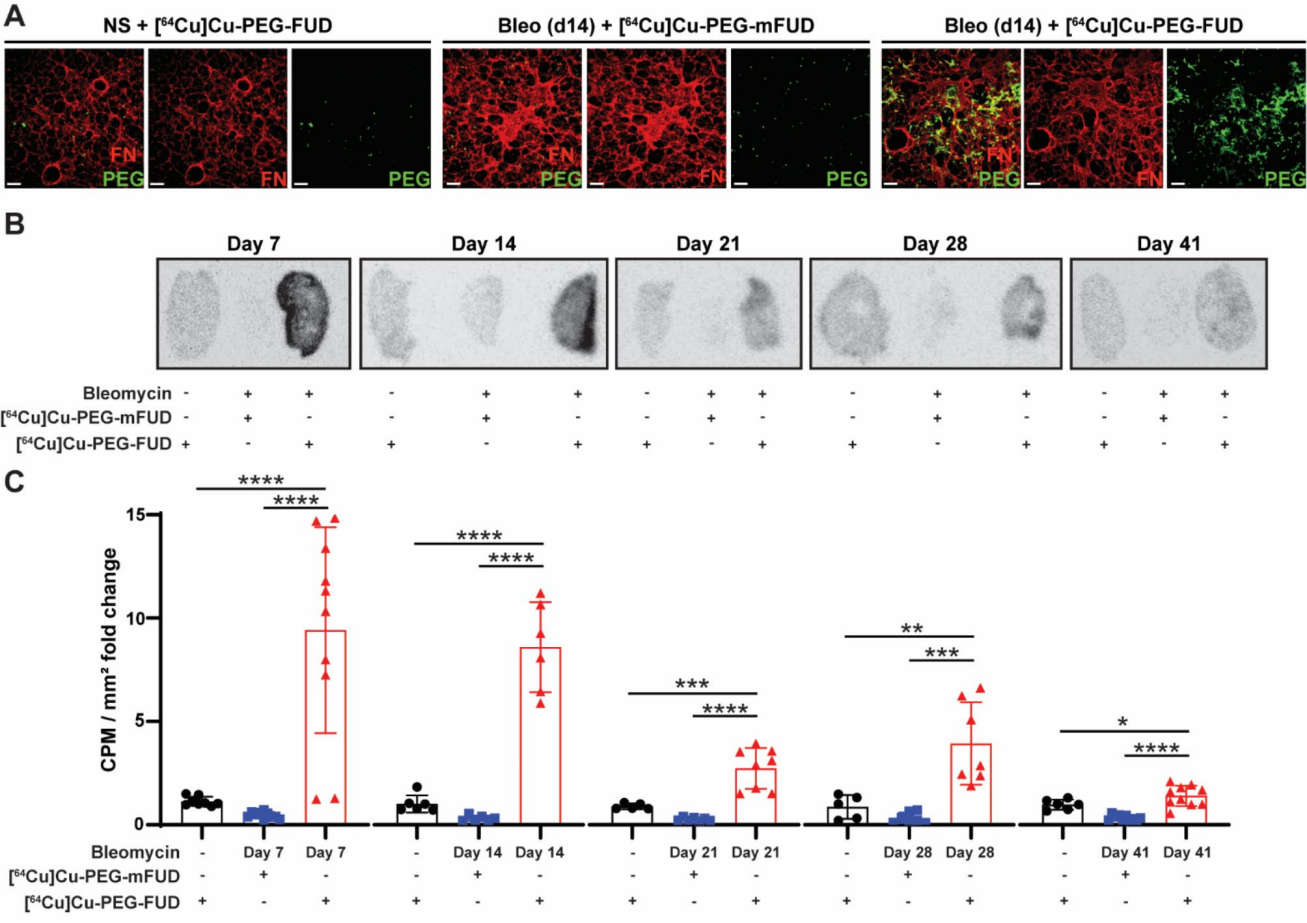
[^64^Cu]Cu-PEG-FUD targeting fibrotic PCLS peaks during the fibrogenic phase of bleomycin-induced pulmonary fibrosis. Mice were intratracheally treated with bleomycin (2 U/kg, IT) or normal saline control. Mouse lungs were sectioned into precision cut lung slices (PCLS) and incubated with radiolabeled peptides ([^64^Cu]Cu-PEG-FUD or [^64^Cu]Cu-PEG-mFUD control). **(A)** PCLS generated 14 days after treatment with bleomycin or normal saline (NS) were stained against fibronectin (FN) and PEG. Scale bar = 100 μm. **(B)** Autoradiography was performed and representative images are displayed with signal adjusted based on normal saline (NS) + [^64^Cu]Cu-PEG-FUD controls at each time point. **(C)** Radioactivity on PCLS was quantified via gamma counting. Counts per minute (CPM) were divided by the area and normalized based on NS + [^64^Cu]Cu-PEG-FUD control signal at each time point. *n* ≥ 2 mice/ NS groups and *n* ≥ 2 slices/mouse NS groups. *n* ≥ 3 mice/ bleomycin groups and *n* ≥ 1 slices/mouse in bleomycin groups. Data are represented as mean ± SD

Our study was designed to determine the binding capacity of our FN-targeting peptide, PEG-FUD, over the course of bleomycin-induced pulmonary fibrosis (Fig. 2). Based on our previous findings with IPF tissue ex vivo, we hypothesized that PEG-FUD may target the fibrogenic phase in this model [19]. Thus, we first prepared and analyzed the PEGylated peptide, followed by radiolabeling with ^64^Cu, to quantify its uptake ex vivo in PCLS derived from mice at different phases of the bleomycin-induced model. Second, we performed single dose toxicity studies, ensuring that our peptide did not result in any adverse effects. Given the encouraging results from these studies, we finally administered the fluorescently labeled peptide (Cy5-PEG-FUD) to mice in vivo, followed by quantification of uptake in mouse lungs, as well as other relevant organs during the bleomycin model. Mice were randomly selected for group placement. Investigators blinded to the treatment groups selected the samples and performed image analysis.

### Peptide synthesis, characterization and reporter conjugation

FUD peptide and the control mutant FUD (mFUD), with alterations to significantly reduce the peptide’s binding affinity to FN [34], were recombinantly produced using BL21 *E. coli* (DE3) and the pET-ELMER vector, as before [28–30]. Peptide purification was done using fast protein liquid chromatography (FPLC) with a HiTrap Q HP column (Cytiva, Marlborough, MA, USA) producing a single peak for FUD and mFUD. The binding affinity of FUD and mFUD for FN was assessed by competitive enzyme linked immunosorbent assay (ELISA) using 0.5 nM biotinylated FUD (b-FUD) along with varying concentrations of unlabeled FUD and mFUD (2000, 1000, 500, 250, 125, 62.5, and 0 nM) and absorbance was subsequently measured at 405 nm using a microplate reader to quantify the binding affinity, as previously described [27, 30, 35]. Next, the FPLC-purified FUD or mFUD peptides were N-terminally conjugated with a 20-kDa methoxy-PEG-propionaldehyde (NOF America Corporation, White Plains, NY, USA), as previously described [28, 30]. The PEG-conjugated peptides were purified using FPLC and PEGylated peptide identity was confirmed using MALDI-TOF analysis [28, 30]. The concentration of the PEG-FUD and PEG-mFUD conjugates were determined by measuring the absorbance at 280 nm, utilizing the specific extinction coefficients (ε) value for FUD (0.496) and mFUD (0.742). Purified PEGylated peptides were conjugated with appropriate reporters, including p-SCN-Bn-NOTA (NOTA; Macrocyclics, Dallas, TX, USA) or sulfo-Cy5-NHS-ester (Lumiprobe, MD, USA), as previously described [30, 36, 37]. To enable radiolabeling, PD-10 columns (Cytiva, Marlborough, MA, USA) were used to purify NOTA-PEG-FUD from unconjugated NOTA. ^64^Cu was produced using published methods by the University of Wisconsin GE PETtrace Cyclotron [38]. Subsequently, 50–150 µg of NOTA-FUD was mixed with 37–111 MBq (1–3 mCi) of ^64^Cu in 200 µL of 0.1 M sodium acetate buffer (pH 5.5) for 60 min at 37 °C. Radiolabeled peptides were purified using PD-10 columns. The specific activity was approximately 50 µg of peptide per 1 mCi of ^64^Cu. Radio-thin layer chromatography performed at the end of the incubation time demonstrated over 90% of radiochemical yield. For fluorescent labeling, mono-labeled Cy5-PEG-FUD conjugates were isolated by ion-exchange chromatography (FPLC). Concentrations of the Cy5-PEG-FUD and Cy5-PEG-mFUD conjugates were determined by measuring the absorbance at 646 nm.

### Bleomycin-induced pulmonary fibrosis

Animal experiments were approved by the Institutional Animal Care and Use Committee (IACUC) at the University of Wisconsin-Madison (protocol numbers M005823 and M005532), which is accredited by the Association for Assessment and Accreditation of Laboratory Animal Care. Male and female mice (8–15 weeks old) on a C57Bl/6J background were anesthetized with ketamine (100 mg/kg; Zoetis Inc., Parsippany-Troy Hills, NJ, USA) and xylazine (15 mg/kg; Akorn Pharmaceuticals, Lake Forest, IL, USA) by intraperitoneal injection and administered a single intratracheal (IT) dose of bleomycin (2 U/kg, Hospira, Lake Forest, IL, USA was used for PCLS studies and 1 U/kg TEVA, Tel Aviv, Israel was used for the remaining studies) in 50 µL of 0.9% normal saline irrigation (NS; Baxter, Madison, WI, USA) or NS alone for non-injured controls, as we have done before [19, 39–41]. Mice were caged separately corresponding to experimental group. At experimental end-point, mice were anesthetized via intraperitoneal delivery of ketamine (200 mg/kg) and xylazine (30 mg/kg) and exsanguinated prior to tissue collection.

### Precision cut lung slices

PCLS processing and analysis was performed based on a protocol we previously developed [19]. Briefly, bleomycin or NS-treated mouse lungs were inflated with warm low melt agarose (IBI Scientific, Dubuque, IA, USA) in phenol-red-free Dulbecco’s modified Eagle’s medium (DMEM; Corning Inc., Corning, NY, USA) supplemented with antibiotics (Penicillin-Streptomycin-Amphotericin (PSA), Corning Inc.: streptomycin (100 µg/ml), amphotericin B (250 ng/ml), penicillin (100 U/ml), 2 mM l-glutamine (Corning Inc.), and 10% fetal bovine serum (HyClone, Buckinghamshire, UK). Lungs in ice-cold supplemented phenol-red-free DMEM media were sequentially sectioned, and subsequently washed at 37 °C prior to incubation with 5 µCi of [⁶⁴Cu]Cu-PEG-FUD (or [⁶⁴Cu]Cu-PEG-mFUD) for a final peptide concentration of 25 nM at 37 °C for 1 h and 45 min. After incubation, slices were washed and subjected to gamma counting using the WIZARD2 (PerkinElmer, Shelton, CT, USA) and then mounted on glass slides for autoradiography (PerkinElmer). Gamma counts per minute (CPM) were divided by the area of each PCLS manually outlined in ImageJ and subsequently normalized to the normal saline + [⁶⁴Cu]Cu-PEG-FUD control condition. At each time point, after ensuring normal distribution, signal from different groups was compared using the One-Way ANOVA with Šídák post-hoc test. PCLS were fixed in 4% paraformaldehyde for 20–30 min, washed and stored in PBS at 4 °C before immunofluorescence staining.

### Tissue imaging

Imaging of relevant organs following in vivo administration of Cy5-PEG-FUD or Cy5-PEG-mFUD was performed in the University of Wisconsin-Madison Carbone Cancer Center Small Animal Imaging and Radiotherapy Facility via the In Vivo Imaging System (IVIS, Spectral Instruments Imaging, Tucson, AZ, USA), as we have previously done [29]. Injection mixture of 1.5% Cy5-labeled PEGylated peptide and 98.5% of corresponding unlabeled PEGylated peptide, at a mass dose of 12.5 mg/kg (52.08 nmol) of peptide equivalent was subcutaneously delivered to separate cohorts of mice 3-, 7-, 14-, 21-, 28-, or 42-days post IT bleomycin or NS administration. One day later, mouse organs, including blood, heart, lung, kidney, and liver, were harvested prior to IVIS imaging. Imaging conditions included: excitation of 640 nm and emission of 680 nm, medium binning, 2 F/Stop and 22.4 cm field of view were used for each imaging session with epi-fluorescence scale bars normalized to the same intensity (5 × 10^7^ to 5 × 10^8^). Images were analyzed using Living Image Software (PerkinElmer) by manually outlining each organ and quantifying total radiant efficiency. At each time point, normality was tested via the Kolmogorov-Smirnov test and signal from each organ was compared between different groups using the One-Way ANOVA with Šídák post-hoc test (following log10 transformation for non-normally distributed data).

### Histology

Lungs collected from mice administered Cy5-PEG-FUD or Cy5-PEG-mFUD control were inflated with 10% formalin (Fisherbrand, Pittsburg, PA, USA) and fixed for 24–48 h before being transferred to 70% ethanol, paraffin embedded, and sectioned. Lung tissue from select mice were subjected to Masson’s trichrome staining (*N* = 1 normal saline/time point and *N* = 4 bleomycin/time point) or staining with anti-FN antibody (RamFN, rabbit polyclonal antibody to mouse FN, diluted at 1:10,000 dilution [42]) and HRP-conjugated secondary antibody (*N* = 3 normal saline total and *N* = 1–2 bleomycin/time point), followed by scanning with the Aperio Digital Pathology Slide Scanner System and digital visualization using Aperio ImageScope (Leica Biosystems, Wetzlar, Germany). As previously described, modified Ashcroft scoring of trichrome stained lung tissue was performed to grade the severity of bleomycin induced pulmonary fibrosis [40, 41, 43]. Briefly, snapshots (*N* ≥ 68 per mouse, 20x magnification) systematically covering the lung parenchyma were taken and scored by two blinded observers. Scores were averaged between observers and averaged scores were plotted as a percentage and stratified based on score severity, 0–3 (mild), 4–5 (moderate), and 6–8 (severe). Average score per animal was plotted against ex vivo lung total radiant efficiency. Normality was tested using the Kolmogorov-Smirnov test and non-normally distributed data was normalized through log10 transformation before applying linear regression. Residual plots were then visually examined to check for any violations of model assumptions, such as non-linearity or unequal variance, ensuring the appropriateness of the regression model. Quantification of FN histology was performed by obtaining 0.6X magnification images of each sample, isolating the brown stain using the ImageJ Immunohistochemistry Toolbox plug-in, inverting the image, outlining the lung and measuring integrated density. Integrated density was divided by the total lung area to account for potential differences in lung size, as before [39]. Data was normality tested before linear regression was applied.

Fixed PCLSs previously incubated with radiolabeled peptides were co-immunostained using antibodies against FN (rabbit polyclonal antibody, ab2413, Abcam, Cambridge, UK), PEG (rabbit monoclonal antibody, ab51257, Abcam) and appropriate secondary antibodies followed by fluorescence signal amplification with Tyramide signal amplification substrates (PerkinElmer). Subsequent imaging was done on Leica Thunder Imaging System (Leica Biosystems). The same parameters were used for imaging all of the conditions and subsequent image enhancement.

### Blood analysis

Mice were treated with a single dose of PEG-FUD, PEG-mFUD (1.56, 3.125, 6.25, 12.5, or 25 mg/kg) or equal volume of PBS vehicle administered via subcutaneous delivery. One day later, blood was collected for complete blood count (CBC) and complete metabolic panel via cardiac puncture, aliquoted into either an ethylenediaminetetraacetic acid (EDTA)-coated tube (CBC) or Lithium Heparin-coated tube (chemistry analysis). CBC analysis of whole blood was performed on Zoetis Vetscan HM5 (Zoetis Services LLC, Parsippany, NJ, USA) within eight hours of collection. Plasma was collected by centrifuging heparinized whole blood (4000 RPM for ten minutes at room temperature), loaded into the Preventative Care Profile Plus rotors (Zoetis #10023238) and subjected to analysis via Zoetis Vetscan VS2 (Zoetis Services LLC).

The data analysis was conducted to compare the effects of PEG-FUD, PEG-mFUD, and vehicle treatments across various analytes at multiple dose levels. Incomplete observations (e.g.: analytes with readings below detection in all animals, such as eosinophil and basophil counts) were not analyzed. For each group, the normality of data distribution was assessed using the Shapiro-Wilk test, which was bypassed if a group had fewer than three observations or showed zero variance, followed by Levene’s test for homogeneity of variance between groups and a subsequent Student’s *t*-test. If variances were unequal, Welch’s t-test was used. In cases where the normality assumption was violated, the non-parametric Mann-Whitney U test was applied.

### Statistical analyses

Data are presented as mean ± standard deviation. One Way ANOVA with Šídák post-hoc test was used for comparisons between multiple groups. For correlative analyses, linear regression method was used to fit the data. *P* ≤ 0.05 was considered statistically significant.

## Results

### Synthesis and PEGylation of purified PEG-FUD/ PEG-mFUD peptides

We recombinantly produced FUD and mutated FUD (mFUD) and confirmed expected binding activity (Figure S1A). We subsequently conjugated the peptides with 20 kD PEG, separated the fractions from unreacted peptides, unreacted PEG, and multi-PEGylated peptides to isolate mono-PEGylated FUD or mFUD (Figure S1B). The successful purification of mono-PEGylated FUD was confirmed by MALDI/TOF mass spectrometry (Figure S1C). In all subsequent studies, we utilized mono-PEGylated FUD or mFUD (referred to as PEG-FUD and PEG-mFUD hereafter), whether alone or conjugated with corresponding reporters, which are anticipated to retain nanomolar affinity for FN, as previously demonstrated by our team [28, 30].

### Temporal binding of [⁶⁴Cu]Cu-PEG-FUD to fibrotic mouse PCLS

**Fig. 3:**
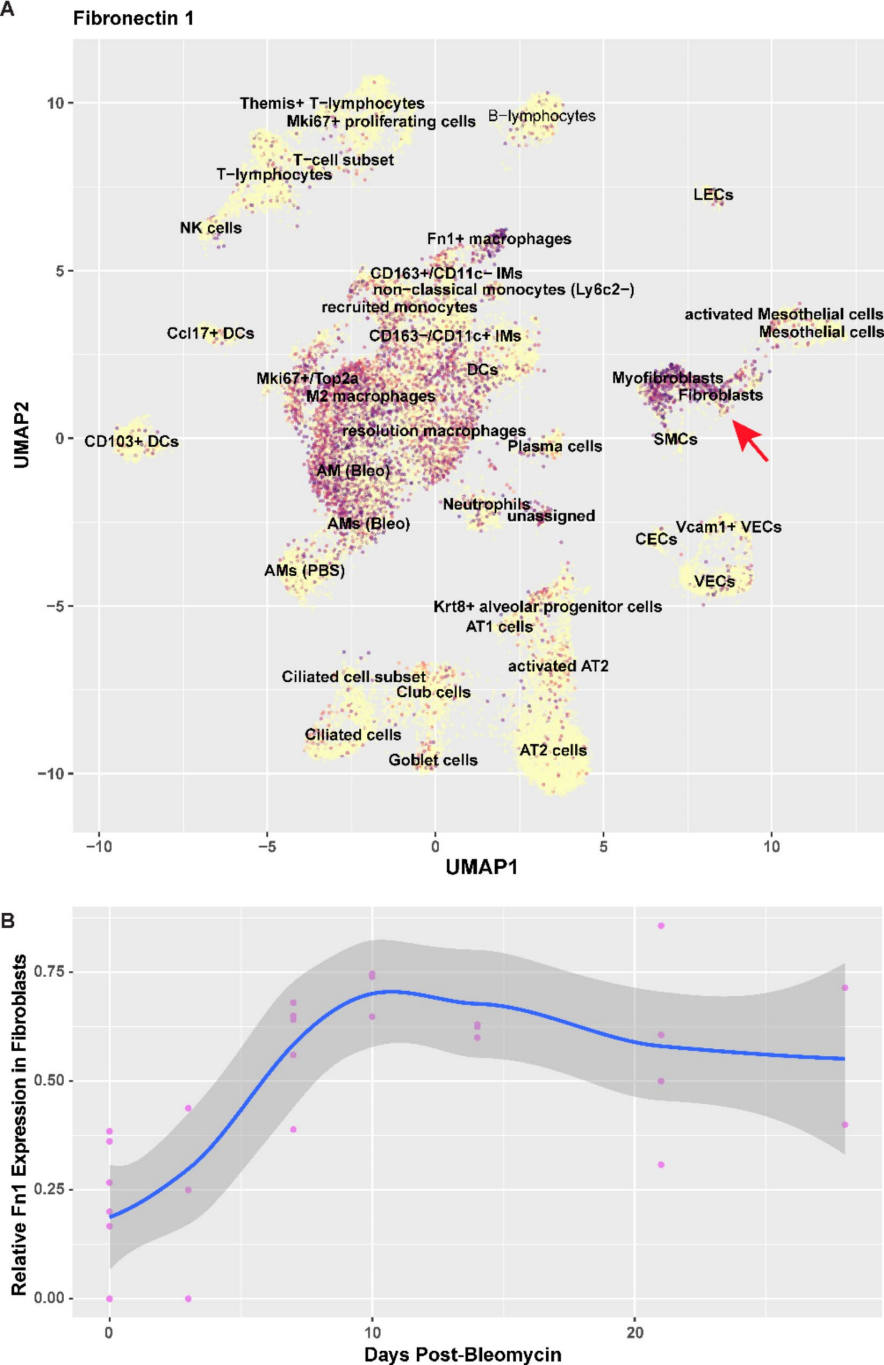
Fibronectin expression in bleomycin-induced lung fibrosis. Single cell RNA sequencing data set taken from the Gene Expression Omnibus interactive webtool by Strunz and colleagues [43]. **(A)** Fibronectin expression in the whole lung cell type signature with red arrow pointing to ECM-mediating fibroblasts/myofibroblasts. **(B)** Fibronectin expression in fibroblasts using whole lung time course differential expression (Spline regression *p* = 0.032)

The bleomycin model of pulmonary fibrosis is characterized by distinct temporal phases, including early injury and inflammation (~ day 2–7), leading to fibrogenesis (~ day 7–14) that is followed by a phase of established fibrosis (~ day 28) and subsequent resolution. Recently, Strunz and colleagues examined the single-cell lung transcriptome over the course of bleomycin-induced pulmonary fibrosis [44]. Using their publicly available interactive web tool, we found that FN expression in fibroblasts, cells responsible for depositing FN into ECM, peaks around day 10 post-bleomycin injury (Fig. 3) [44]. These findings are consistent with other studies that show the peak of FN matrisome within a similar period post-bleomycin injury [45].

We have previously shown that [⁶⁴Cu]Cu-PEG-FUD preferentially binds to fibrotic mouse PCLS eleven days following bleomycin instillation, during the phase of extensive fibrogenesis [19]. However, it is not known how uptake of [⁶⁴Cu]Cu-PEG-FUD changes with respect to each of the distinct phases of bleomycin-induced injury and fibrosis. Thus, we initially sought to investigate the temporal changes in [⁶⁴Cu]Cu-PEG-FUD binding to mouse PCLS isolated from discrete timepoints over the course of bleomycin-induced model of pulmonary fibrosis. Freshly isolated mouse PCLS from different phases of the model were incubated in PCLS culture media for 105 min with [⁶⁴Cu]Cu-PEG-FUD, followed by autoradiography and gamma counting. To confirm appropriate peptide targeting, a subset of PCLS isolated 14 days post-bleomycin administration were fixed in 4% paraformaldehyde and co-stained against the PEG portion of PEG-FUD and FN, the peptide target. Our staining revealed increased PEG staining that overlaps with FN deposition (Fig. 2A) in bleomycin-treated lungs incubated with [⁶⁴Cu]Cu-PEG-FUD, suggesting that PEG-FUD peptide targeted to fibrotic lungs ex vivo. On the other hand, we observed little PEG staining in the PCLS from uninjured lungs incubated with [⁶⁴Cu]Cu-PEG-FUD or fibrotic lungs incubated with the control peptide, [⁶⁴Cu]Cu-PEG-mFUD. We utilized autoradiography and gamma counting to visualize and quantify the amount of bound radiolabeled peptide at each timepoint. We found that [⁶⁴Cu]Cu-PEG-FUD bound more readily to the fibrotic PCLS compared to uninjured PCLS at each time point analyzed, including days 7, 14, 21, 28, and 41 post-bleomycin (Fig. 2B, C). Moreover, we confirmed that at each of these time points, [⁶⁴Cu]Cu-PEG-FUD maintained significantly higher binding capacity compared to the mutant control, [⁶⁴Cu]Cu-PEG-mFUD, further corroborating PEG-FUD’s specificity to its target, FN, an important component in the fibrotic process [21, 24]. Additionally, by comparing [⁶⁴Cu]Cu-PEG-FUD binding to PCLS during different time points post-bleomycin, we found that the highest level of targeting to fibrotic PCLS occurred 7–14 days post-bleomycin, representing the timing of extensive fibrogenesis. Overall, these data show that [⁶⁴Cu]Cu-PEG-FUD targets PCLS from bleomycin-treated lungs, peaking at the time points characterized by *de novo* ECM deposition in the early phase of active disease.

### Single dose effects of PEG-FUD

To determine preliminary metrics of safety for use in vivo, we examined the potential toxicity of PEG-FUD in mice. We reviewed mouse health and several metrics of metabolic health, including blood counts and plasma indices of renal and hepatic health 24 h post-subcutaneous injection of various doses of PEGylated peptides (PEG-FUD or PEG-mFUD) or vehicle control. Mice appeared to have no behavioral or overall health change after one administration of PEG-FUD at any of the doses tested (0.16 mg/kg to 25 mg/kg). As demonstrated in Table S1, we observed significant differences in several indices of RBC shape and composition, including mean corpuscular volume (MCV), hematocrit (HCT), mean corpuscular hemoglobin concentration (MCHC), and red blood cell distribution (RDW) compared to vehicle control (Figure S2A-D). We also found changes in blood urea nitrogen (BUN), a marker of kidney function (Figure S2E). However, for all of these analytes the effect size was small and not clinically significant with the observed differences being largely between vehicle control and both peptides (both PEG-FUD and PEG-mFUD), indicating a potential role for the PEG moiety. In addition, we found some significant changes in the markers of liver function, such as alanine transaminase (ALT), total protein (TP) and albumin (ALB) (Figure S2F-H). Despite the liver hepatocytes being one of the major producers of liver FN, the presented changes did not appear specific to the FN-targeting peptide, nor did they trend with increasing dose of the peptides. Altogether, these findings did not show any clinically relevant effects of PEG-FUD on animal health. These data are consistent with previous studies using PEG-FUD and FUD in mouse models of cardiac, kidney, and liver fibrosis without any reported adverse effects [46–50].

### Temporal binding of Cy5-PEG-FUD in relevant mouse organs

Given that our data supported the safety of a single bolus dose of PEG-FUD in vivo, we sought to assess the peptide biodistribution in relevant organs. For this, separate mouse cohorts were subcutaneously administered fluorescently-labeled Cy5-PEG-FUD (or Cy5-PEG-mFUD control) and the total radiant efficiency in the blood, heart, lung, liver, and kidney ex vivo were quantified the following day using the IVIS fluorescence imaging. In healthy mice, analysis 24 h post-injection revealed continued circulation of fluorescently-labeled Cy5-PEG-FUD in the blood (2.31 × 10^8^ [p/s] / [µW/cm^2^]) and heart (3.14 × 10^8^ [p/s] / [µW/cm^2^]). We also detected peptide retention in the lung (2.76 × 10^9^ [p/s] / [µW/cm^2^]), liver (4.22 × 10^9^ [p/s] / [µW/cm^2^]) and kidney (2.54 × 10^9^ [p/s] / [µW/cm^2^]).

**Fig. 4:**
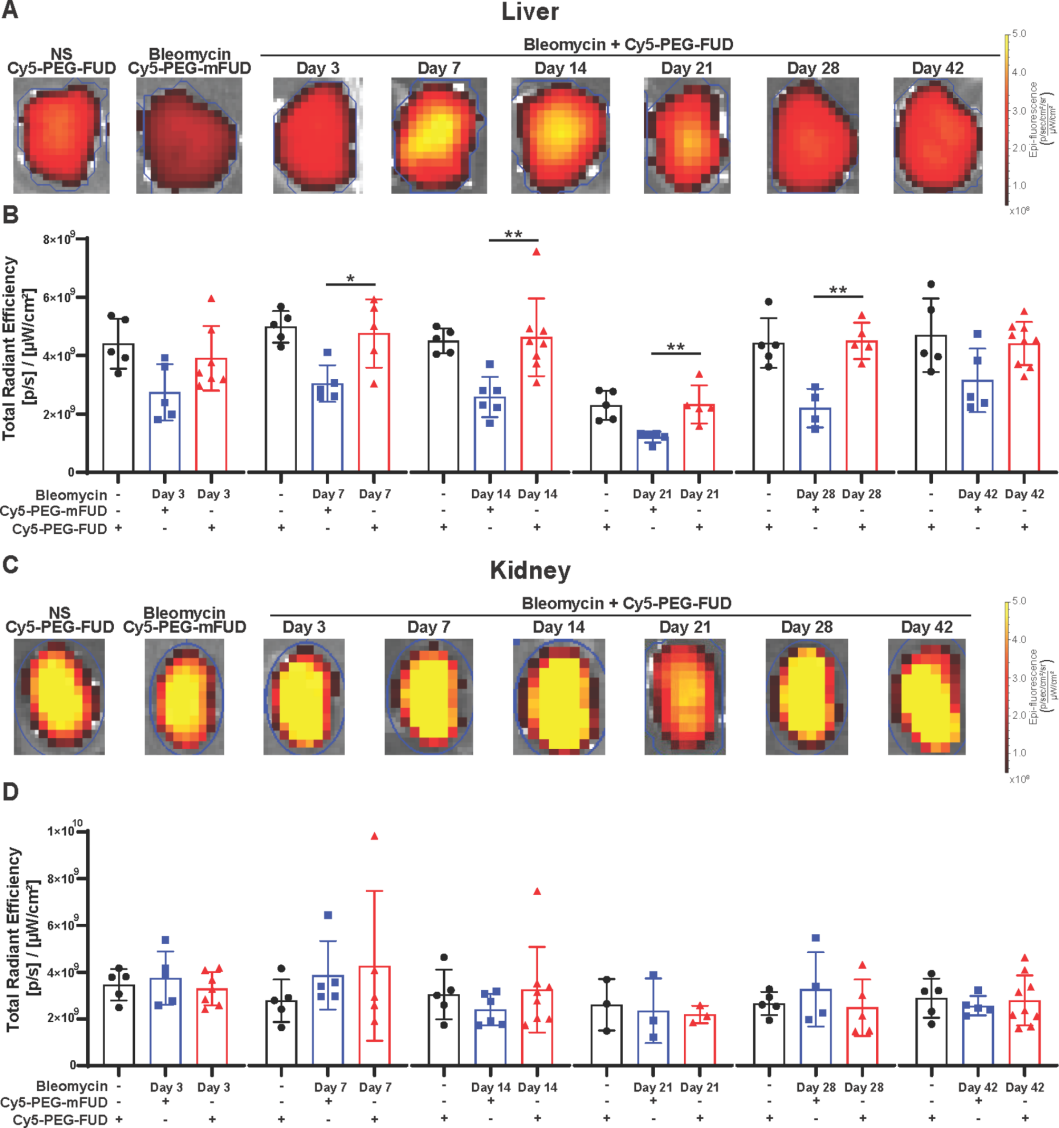
Cy5-PEG-FUD uptake is increased in liver compared to mutant control, while both peptides appear to be renally cleared. Mice were treated intratracheally with bleomycin (1 U/kg, IT) or normal saline. At indicated time points, Cy5-PEG-FUD or Cy5-PEG-mFUD (0.1875 mg/kg Cy5-labeled peptide in 12.5 mg/kg mass dose) were administered subcutaneously. One day later, liver (**A**,** B**) and kidney (**C**,** D**) tissues were collected. **(A)** Representative images of livers are included. **(B)** Total radiant efficiency was quantified in livers. **(C)** Representative images of kidneys are included. **(D)** Total radiant efficiency was quantified in kidneys. *n* ≥ 3 mice/group. Data are represented as mean ± SD

Subsequently, we assessed the uptake of Cy5-PEG-FUD and Cy5-PEG-mFUD control in relevant extrapulmonary organs/compartments at several timepoints after delivery of a single dose of intratracheal bleomycin. Analysis of the blood, heart, and liver revealed that Cy5-PEG-FUD uptake was higher than that of Cy5-PEG-mFUD at most timepoints, likely due to Cy5-PEG-FUD binding to FN in those tissues (Fig. 4 and S3). However, we found no difference in uptake in these organs between healthy mice and those subjected to bleomycin treatment at any of the time points. Notably, in the kidney Cy5-PEG-FUD and Cy5-PEG-mFUD peptide uptake was the same (Fig. 4), likely due to the peptides’ propensity for renal clearance in both healthy and bleomycin-injured mice [19, 29]. Altogether, these studies demonstrate that uptake of Cy5-PEG-FUD in the heart, blood, liver, and kidney is unchanged in healthy mice and throughout the course of bleomycin-induced lung fibrosis.

### Temporal binding of Cy5-PEG-FUD in fibrotic mouse lungs

**Fig. 5:**
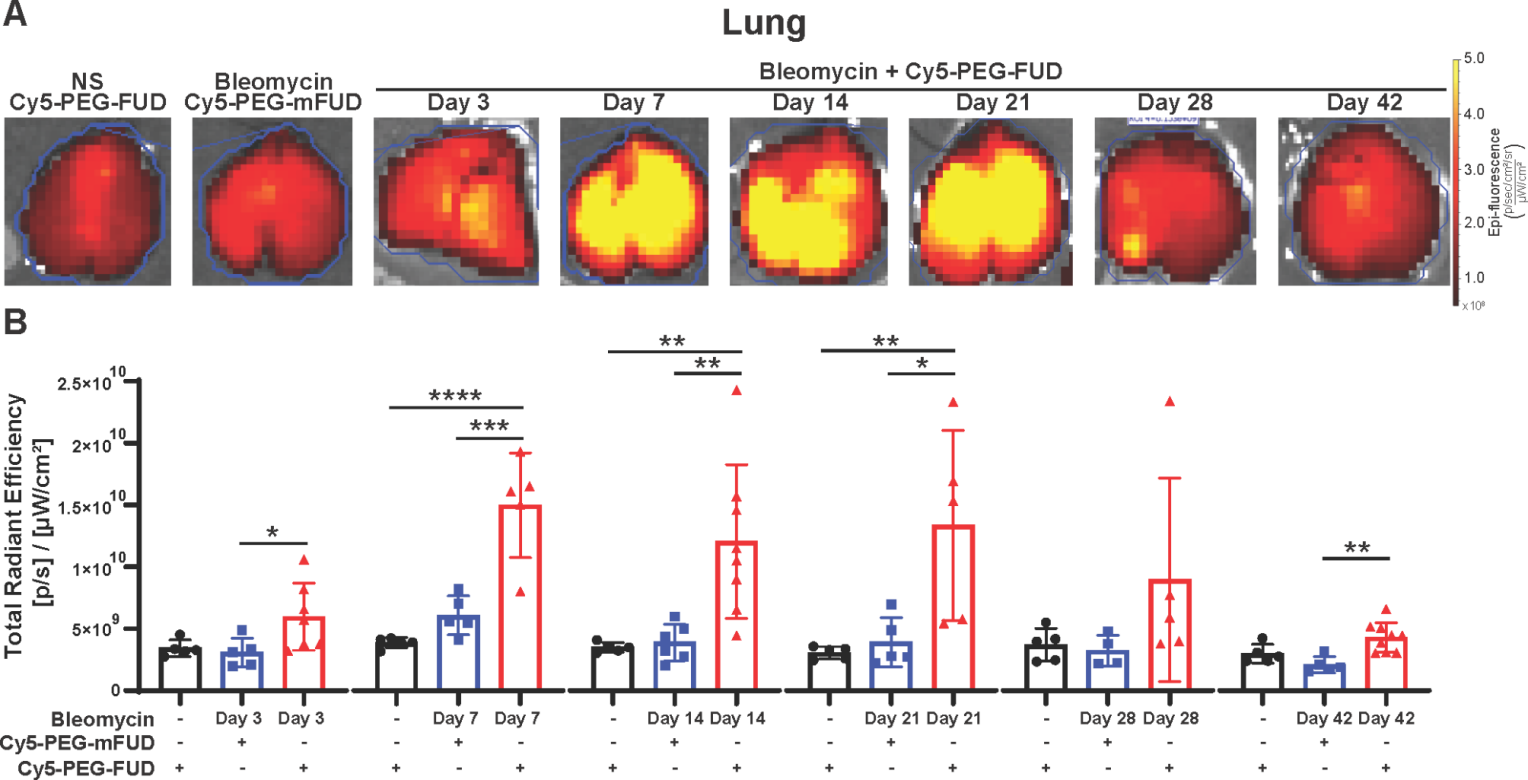
Cy5-PEG-FUD targets the fibrogenic phase of the bleomycin-induced pulmonary fibrosis in mice. Mice were treated with intratracheal bleomycin (1 U/kg, IT) or normal saline control. Cy5-PEG-FUD or Cy5-PEG-mFUD control (0.1875 mg/kg Cy5-labeled peptide in 12.5 mg/kg mass dose) were subcutaneously administered to different groups of mice at indicated time points, followed by ex vivo lung tissue imaging 24 h later using the In Vivo Imaging System. **(A)** Representative images of lung tissue from each group are included. **(B)** Total radiant efficiency was quantified and compared at each time point using One Way ANOVA with Šídák post-hoc test. *n* ≥ 3 mice/group. Data are represented as mean ± SD

Since there was a differential uptake of [⁶⁴Cu]Cu-PEG-FUD by fibrotic PCLS during various timepoints in the bleomycin model of pulmonary fibrosis, we were interested in assessing the probe’s uptake in bleomycin-injured lungs after subcutaneous administration during different phases of this model. On days 3, 7, 14, 21, 28, and 42 post-bleomycin, separate mouse cohorts were administered Cy5-PEG-FUD (or Cy5-PEG-mFUD). The following day, mouse tissues were isolated and imaged ex vivo. Lung image analysis revealed increased Cy5-PEG-FUD lung uptake 7, 14, and 21 days post-bleomycin in comparison to both control groups: fibrotic lungs from mice administered Cy5-PEG-mFUD control (at equivalent time points) and normal lungs from mice administered Cy5-PEG-FUD (Fig. 5). Cy5-PEG-FUD uptake in lungs treated with bleomycin 3 and 42 days earlier was only higher compared to the uptake of Cy5-PEG-mFUD control but not compared to normal lungs with Cy5-PEG-FUD. Interestingly, there was no difference in uptake between any of the conditions 28 days post-bleomycin. Overall, these data show approximately a 4-fold increase of PEG-FUD probe uptake in the bleomycin-treated lungs during the fibrogenic and early established fibrosis phase (day 7–21) compared to normal controls, while the uptake during phases characterized by inflammation (day 3), established fibrosis (day 28), and resolution (day 42) was only 2-fold higher compared to normal controls. Importantly, select tissue samples from mice throughout the course of the bleomycin-induced model were stained against FN, where we detected a correlative relationship between FN expression and Cy5-PEG-FUD uptake in the lungs (Figure S4). This finding supports the potential for PEG-FUD to target FN throughout the course of the model.

### Correlation of PEG-FUD uptake with fibrotic burden

**Fig. 6:**
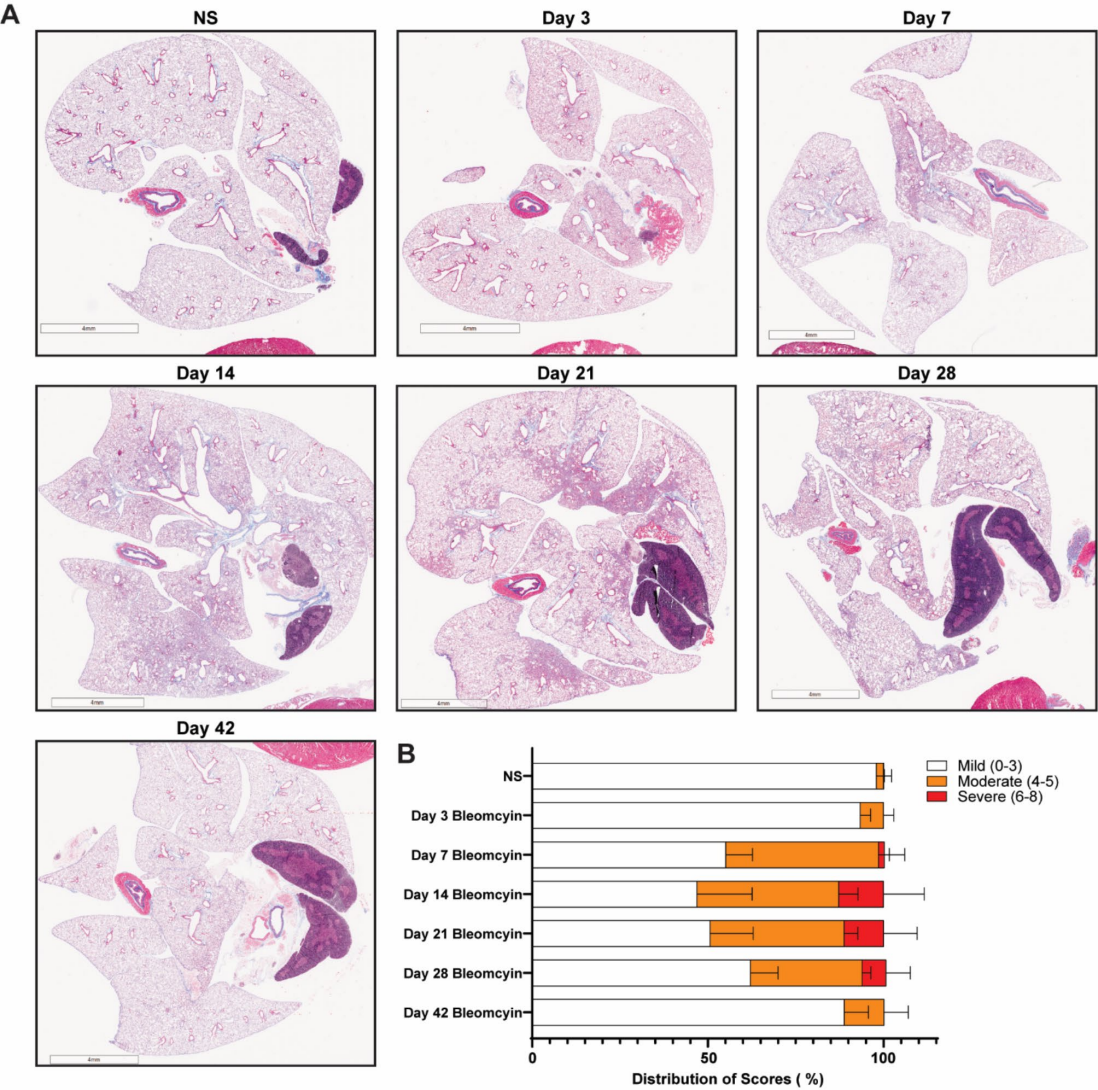
Trichrome staining and Ashcroft scoring reveal the pattern of fibrotic burden during the bleomycin-induced murine model of pulmonary fibrosis. Mice were treated with bleomycin (1 U/kg, IT) or normal saline control followed by lung tissue collection. Masson’s trichrome staining (**A**) and Ashcroft scoring (**B**) was performed at indicated time points. Scale bar = 4 mm. Data are represented as mean ± SD

Next, to compare PEG-FUD uptake with overall fibrotic burden at each time point, we quantified fibrosis in a subset of mouse lung sections from the Cy5-PEG-FUD cohort above by Masson’s trichrome staining, followed by modified Ashcroft scoring [43]. We found that the Ashcroft scores fit within the patterns of expected fibrotic development in the bleomycin-treated mice over time, with mild fibrotic scores predominating during the early pro-inflammatory phase (day 3 post-bleomycin) and during the resolution phase (day 42 post-bleomycin). Moderate and severe fibrosis were present during the fibrogenic phase (day 7–14) and established fibrosis (day 21–28) (Fig. 6). 

**Fig. 7:**
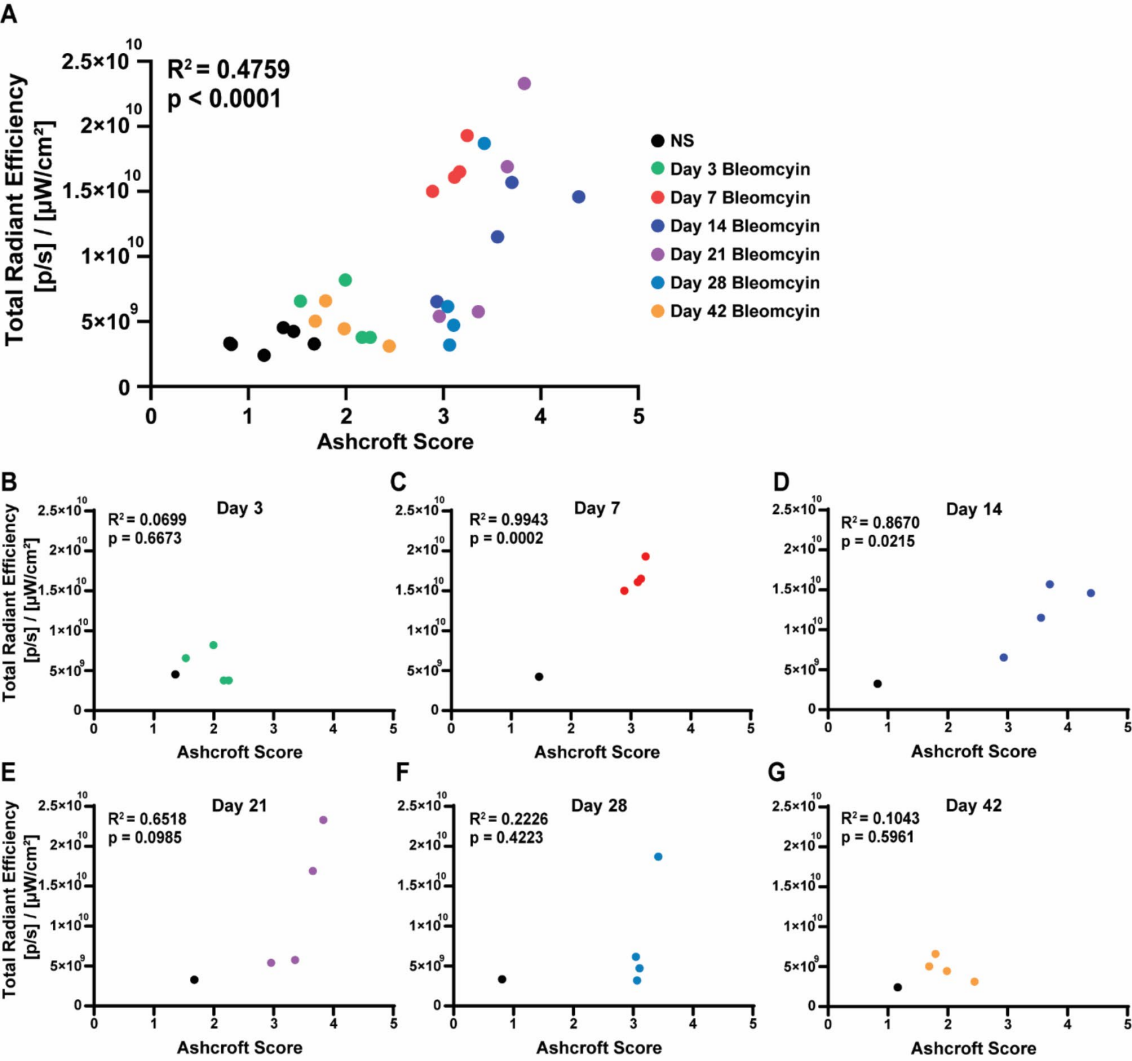
Cy5-PEG-FUD uptake correlates with fibrosis during the fibrogenic phase of bleomycin-induced pulmonary fibrosis in mice. Mice were treated with bleomycin (1 U/kg, IT) or normal saline before subcutaneous administration of Cy5-PEG-FUD in PEG-FUD (0.1875 mg/kg Cy5-PEG-FUD in 12.5 mg/kg mass dose). One day later, mouse lung tissues were imaged ex vivo using the In Vivo Imaging System and total radiant efficiency quantified. Subsequently, the same tissues were subjected to Masson’s trichrome staining and modified Ashcroft scoring to quantify fibrosis. The total radiant efficiency and Ashcroft scores from each lung were plotted and linear regression modeling was performed on log10 transformed, normalized data combined for all time points (**A**) and for indicated time points (**B-G**)

We then determined whether Cy5-PEG-FUD lung uptake correlated with the degree of fibrotic burden. First, we detected a significant positive correlative relationship between Cy5-PEG-FUD lung uptake and fibrotic burden by Ashcroft score when considering all timepoints together and including uninjured lungs due to their non-zero presence of fibronectin (Fig. 7A and S4B). Given the temporal evolution of this model, exemplified by different transcriptome, proteome and matrisome profiles during these unique time points in the model, we were interested in examining the relationship between Cy5-PEG-FUD uptake and fibrosis at each time point (Fig. 7B-G). The linear regression analysis demonstrated the strongest correlative relationship between total radiant efficiency resulting from Cy5-PEG-FUD uptake in the lung compared to fibrosis in samples from days 7 and 14 (Fig. 7C-D). On the other hand, the relationship between Cy5-PEG-FUD uptake and fibrotic scoring was not significant on days 3, 21, 28, and 42. Taken together, this provides evidence that Cy5-PEG-FUD may be most suited to target the early fibrogenic phase in the bleomycin model.

## Discussion

FN is an ECM glycoprotein essential for deposition of the ECM components during lung fibrosis and is correlated with clinical parameters of disease progression [6, 21, 22, 24]. Due to its critical role during early lung fibrosis disease development, we examined a FN-targeting peptide, PEG-FUD, as a probe for real-time assessment of disease progression [19]. In this study, we identified the localization of PEG-FUD to the fibrotic lung throughout the course of bleomycin-induced murine pulmonary fibrosis. We show that PEG-FUD peptide uptake increases during the fibrogenic phase of this model, confirming that this peptide could be an excellent candidate for non-invasive monitoring of pro-fibrotic activities.

Our previous studies examining the binding of PEG-FUD in the spatially heterogenous human IPF lung ex vivo demonstrated that this peptide was found preferentially in areas of nascent fibrosis, such as the fibroblastic foci [19]. In turn, we found that areas characterized by end-stage fibrosis with mature scarring showed lower levels of peptide uptake, suggesting that PEG-FUD may specifically target nascent regions of fibrosis, consistent with the spatial profiling of FN expression in the IPF lung [24]. In this work, we assessed peptide targeting in the single-dose intratracheal bleomycin model of murine pulmonary fibrosis [45, 51, 52]. This is a well-established model of self-resolving lung fibrosis that exhibits temporally distinct phases, with upregulation of FN expression in the mesenchyme peaking in the second week post-bleomycin (Fig. 3) [44, 45, 53]. Using this model, we analyzed the level of peptide binding through two separate methods. First, we analyzed direct binding of the radiolabeled peptide, [^64^Cu]Cu-PEG-FUD (or corresponding control [34]), to fibrotic PCLS over the course of this model in comparison to normal PCLS. We found that [^64^Cu]Cu-PEG-FUD more readily targeted the fibrotic PCLS at all points in the bleomycin-induced model compared to the controls, with peak uptake found during the day 7–14 post-bleomycin timepoints, corresponding to expected FN expression in this model [44, 45, 53]. To examine these results in further detail and considering our previous work extensively profiling the absorption kinetics of fluorescently labeled peptide, Cy5-PEG-FUD, following subcutaneous delivery, we administered fluorescently labeled peptides subcutaneously to mice in vivo [29, 30]. We subsequently quantified its targeting to the relevant organs ex vivo, including lungs, after bleomycin instillation. Since the mechanisms of delivery differed between PCLS and in vivo administration, this likely led to differential availability of peptide for binding in vivo due to FN targeting in additional tissue, including the liver [19, 54], as well as systemic circulation and clearance. Still, we found that patterns of lung uptake were similar between the two models. Consistent with our PCLS data, in vivo administration resulted in the highest differential uptake compared to non-injured lungs 7–21 days post-bleomycin, during the phases of fibrogenesis and early established fibrosis. This is supported by the correlative relationship between Cy5-PEG-FUD uptake with histological fibrosis on days 7 and 14.

The propensity of PEG-FUD to target nascent fibrotic processes may be due to several factors. First, FN transcriptome and matrisome expression peaks in the early phase of the bleomycin-induced lung fibrosis (~ day 10 post-bleomycin, Figs. 3 and [44, 45]), following a comparable pattern to that demonstrated in our studies. Similarly, in human disease FN is highly and differentially upregulated in fibroblastic foci, in contrast to other regions of the fibrotic lung, including the mature scar [24]. Second, given the localization of ECM-binding sites within the FN fibril, upon deposition of FN and other ECM molecules into the FN scaffold, the ability of PEG-FUD peptide to bind to FN’s 70kD terminal may be compromised by collagen binding to FN [22, 25, 35]. Finally, it has been previously reported that FN is kept at high tension in healthy tissues, relaxing under pathologic conditions [55]. Thus, it is possible that PEG-FUD peptide exhibits differential kinetics for binding FN under the more relaxed tensional state in fibrosis. Altogether, these findings are in line with our previous studies utilizing clinical lung tissue which suggest that PEG-FUD targets FN more readily during *de novo* ECM deposition rather than the extensively remodeled, end-stage fibrosis [19].

We previously determined that the conjugation of FUD to single 20 kD linear PEG group increases the systemic circulation [28, 29]. This is evident through the continued presence of Cy5-PEG-FUD in the blood and heart 24 h post-injection (Figure S3). While our assessment of blood markers did not reveal effects specific to the FN-targeting peptide, we noted changes in the profile of select blood markers from PEGylated molecules compared to the vehicle control. Specifically, PEG-FUD and PEG-mFUD may have an impact on red blood cells (RBC), potentially indicative of increased osmolality, which has been previously reported with PEGylated compounds [56, 57]. Given its critical importance in homeostatic physiology, we were interested in assessing the potential effect of PEG-FUD administration on other relevant organ function. In the liver, Cy5-PEG-FUD uptake was increased, likely due to the hepatocytic plasma FN synthesis (Fig. 4A, B), while the high level of uptake of both peptides in the kidney suggests renal clearance, as we have previously observed [19]. Finally, the metabolic panel revealed some statistically significant changes (Table S1 and Figure S2E-H). However, these changes did not appear to be clinically relevant as the analytes from mice treated with PEGylated molecules remained within or near the normal range and PEGylated compounds are routinely used clinically [58, 59].

Important limitations of the study include the use of the bleomycin model of murine pulmonary fibrosis, which is commonly used for in vivo assessments of therapeutic and imaging agents [12, 17, 19, 20, 41, 52, 60]. Despite its importance to the field of lung fibrosis research, this model does not perfectly recapitulate human disease as it lacks histopathologic features, including the fibroblastic foci, and is self-resolving in contrast to the human disease. Thus, this model may exhibit different uptake dynamics compared to human disease or other animal models. In addition, administration of bleomycin results in an inflammatory phase prior to the onset of fibrosis, which is qualitatively different from many human ILDs, including IPF. However, this model does recapitulate important features of fibrotic development, including recapitulation of aberrant cell phenotypes found in human disease, centrality of TGF-β signaling in fibrotic progression, and similar ECM compositional changes, including FN deposition. Additionally, this model results in increased vascular permeability during these early phases of the model which can confound molecular probe uptake [61]. However, we utilized the Cy5-PEG-mFUD control, which differs from Cy5-PEG-FUD by seven amino acid residues that significantly decrease its capacity for binding FN [34]. We show that while there is some increase in Cy5-PEG-mFUD uptake that may be a result of vascular leakage post-bleomycin, the uptake of Cy5-PEG-FUD is significantly higher than this control even at early time points. Another limitation of this study was the small sample size for each of the individual time point correlative analyses comparing Ashcroft scoring and total radiant efficiency. Still, the residual plots were visualized following analyses to ensure normality of the results. Finally, this study did not involve in vivo imaging. For this study, we elected to demonstrate peptide binding using two separate ex vivo imaging methods. While PCLS were directly incubated with radiolabeled peptides, fluorescently labeled peptides administered subcutaneously in vivo followed by ex vivo tissue imaging enabled us to glean differential organ uptake more closely. Although the end goal is to employ this peptide as a radiolabeled PET probe, for these studies we chose to pursue additional, complementary avenues of characterizing the temporal uptake over the bleomycin-induced model to expand our experimental capabilities. In the future, these results will be validated through PET imaging of radiolabeled peptides following intravenous delivery, as well as in alternative models of lung fibrosis.

This study has potential impact for the field of molecular imaging of lung fibrosis, as methods that permit more precise and intricate assessment of disease progression are needed in clinical practice. In pulmonary fibrosis, potential molecular imaging targets being developed represent various disease mechanisms, such as activity of pathogenic fibroblasts and macrophages, ECM deposition, as well as detection of pro-fibrotic integrins [12, 14, 15, 17, 62–64]. Patterns of peak uptake in the early phase of the bleomycin model that decline during the established phase are seen with certain molecular targets, such as [^18^F]-fluorodeoxyglucose ([^18^F]FDG) PET, which reveals glucose uptake and metabolism, and [^64^Cu]NODAGA-CG34, a peptide reporter of pro-fibrotic monocyte-derived macrophages [9, 13, 65]. However, both of these probes target alternative mechanisms, such as cellular metabolism and inflammation, which may confound its use in clinical practice. We see a similar pattern of peak uptake during bleomycin-induced fibrogenesis by targeting an ECM glycoprotein, FN, which does not temporally overlap with the inflammatory phase of the model suggesting potential specificity for fibrogenesis. In IPF, FN is associated with clinical markers of disease progression and preferentially upregulated in fibroblastic foci, which represent the sites of early fibrogenesis from which fibrosis spreads [6, 23, 24]. Early detection of FN deposition is especially noteworthy since ECM deposition is a key step in the development and progression of fibrosis and thus, detection of early phases of this process can provide critical information about ongoing activity specific to the molecular mechanisms of this disease. For this reason, FN has been considered as an imaging biomarker in pulmonary fibrosis and other disorders marked by ECM deposition, including cancer [20, 30]. Since FN is involved in other important biologic mechanisms, such as wound healing, utilization of molecular imaging to target FN would provide the added benefit of combining additional, advanced imaging approaches, such as HRCT, to enable detailed characterization of the parenchymal abnormalities in parallel. Other ECM and associated molecular imaging targets of pulmonary fibrosis are also being developed, including collagen, collagen oxidation, allysine, all of which have been shown to be increased in the fibrotic lungs [17, 66–68]. PEG-FUD is advantageous because of its nanomolar binding affinity for FN and its potential to detect fibrogenic activity earlier given the critical role that FN plays in disease progression and regulating the deposition of other ECM molecules [6, 22, 69, 70].

In summary, this study interrogated the longitudinal uptake of a FN-targeting peptide, PEG-FUD, in the bleomycin-induced murine model of pulmonary fibrosis. Using both direct incubation of PCLS and indirect targeting through in vivo delivery, we found increased probe uptake during the fibrogenic phase after bleomycin-induced lung injury. This is an important demonstration of an ECM-targeting probe that may be translatable for non-invasive, real-time detection of early disease mechanisms, something currently unavailable but critically needed to provide clinicians the important insight into patients’ disease status and the need for alteration in therapeutic management. To this end, future studies will focus on using PET imaging to confirm these trends of the radiolabeled probe uptake in both resolving and non-resolving preclinical models of lung fibrosis, as well as examine the capacity of this probe to predict future fibrotic burden and monitor antifibrotic treatment response, critical aspects of patient care that cannot be ascertained contemporaneously using currently available clinical methods.

## Electronic supplementary material

Below is the link to the electronic supplementary material.

Supplementary Material 1

## Data Availability

The experimental data that support the findings of this study are available in Figshare with the identifier 10.6084/m9.figshare.27741678.v1.
