# Supplementary material for: The fibronectin-targeting PEG-FUD imaging probe shows enhanced uptake during fibrogenesis in experimental lung fibrosis
Source: Respir Res. 2025 Jan 22;26:34. doi: 10.1186/s12931-025-03107-x (PMC11756063; doi:10.1186/s12931-025-03107-x)
Supplement: Supplementary file 1 — Supplementary Material 1 [file 12931_2025_3107_MOESM1_ESM.docx]

Supplemental Figures


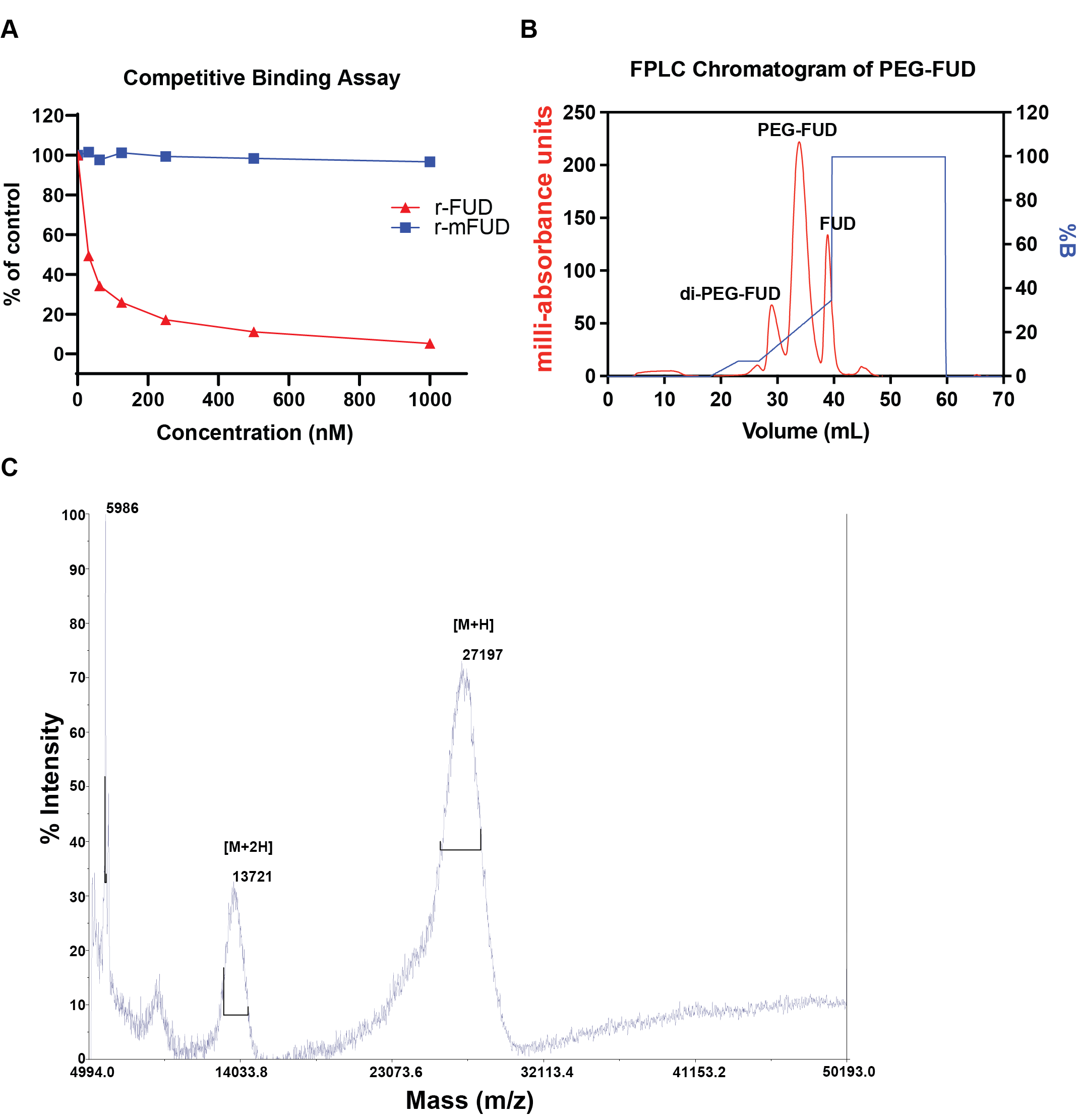


**S1: Characterization and fibronectin-binding of PEG-FUD. A.** Enzyme-linked competitive binding assay showing the percent of b-fud attachment to adsorbed FN when co-incubated with different concentrations of PEG-mFUD or PEG-FUD (n = 3 per group)**.** **B.** FPLC chromatogram of PEG-FUD showing FUD, mono-PEG-FUD (PEG-FUD) and di-PEGylated FUD (di-PEG-FUD) eluted peaks obtained after purification. Side A 20mM Tris A (pH 8.0) and Side B 20 mM Tris + 1 M NaCl. %B = % elution buffer. **C.** MALDI/TOF spectrum to verify FPLC PEG-FUD purification.


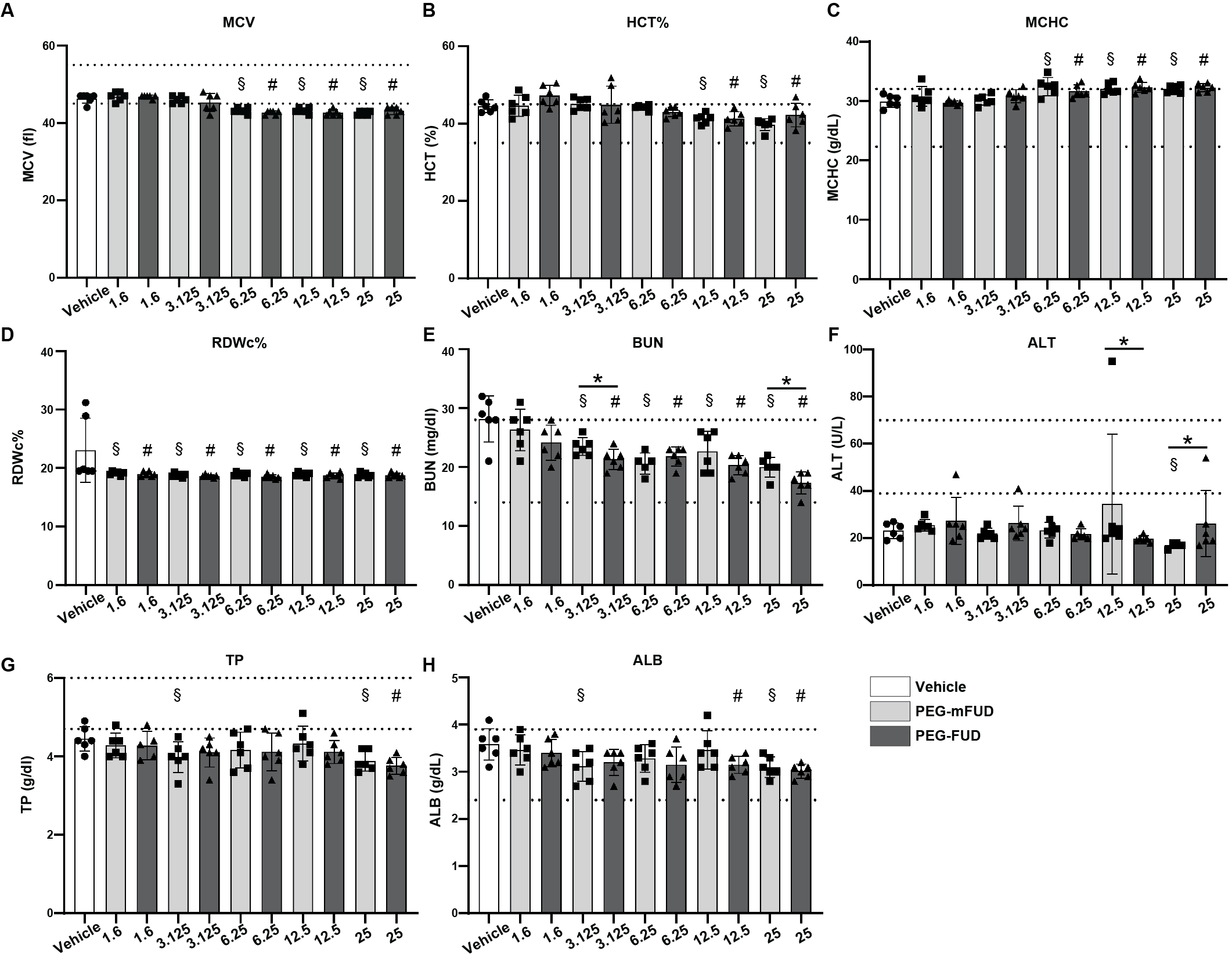


**Figure S2: Blood analysis following escalating single dose peptide administration.**Groups of mice were administered different doses of PEG-FUD, PEG-mFUD or equivalent volume of vehicle control. One day later, mouse blood was collected for complete blood count and complete metabolic panel analysis. Complete blood count analytes, including mean corpuscular volume (MCV, **A**), hematocrit (HCT, **B**), mean corpuscular hemoglobin concentration (MCHC, **C**), and red blood cell distribution (RDW, **D**) were found to have different levels between groups. Complete metabolic panel analytes, including blood urea nitrogen (BUN, **E**), alanine transaminase (ALT, **F**), total protein (TP, **G**) and albumin (ALB, **H**) were found to have differing levels between the groups. Normal analyte ranges are shown with dotted lines. * p < 0.05 between PEG-FUD and PEG-mFUD, # p < 0.05 between PEG-FUD and vehicle, § p < 0.05 between PEG-mFUD and vehicle. n ≥ 5 mice/group. Data are represented as mean ± SD.


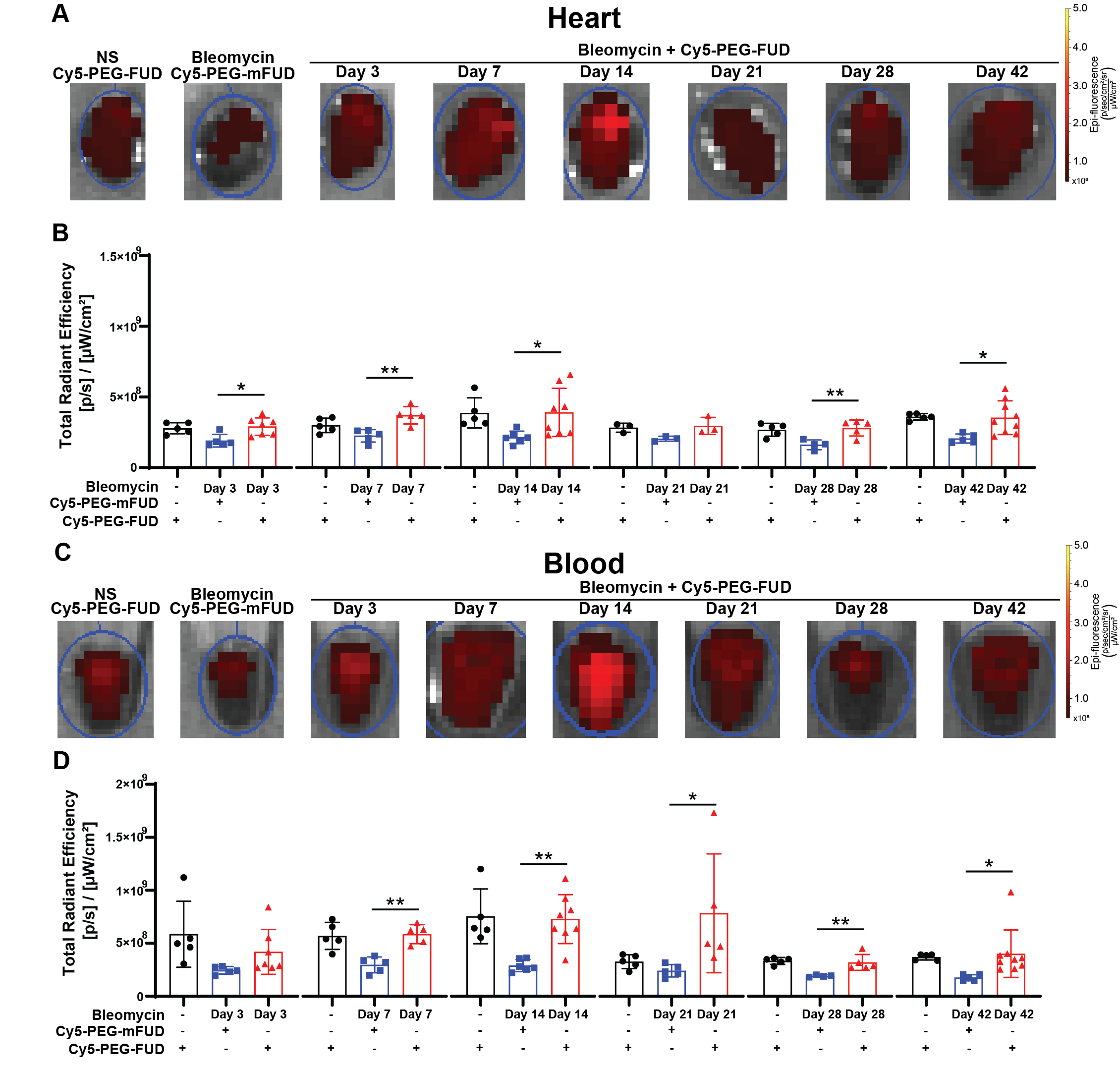


**Figure S3: Heart and blood uptake of Cy5-PEG-FUD.** Mice were treated with bleomycin (1U/kg, IT) or normal saline. At indicated time points, Cy5-PEG-FUD or Cy5-PEG-mFUD (0.1875 mg/kg Cy5-labeled peptide in 12.5 mg/kg mass dose) were administered subcutaneously, followed by *ex vivo* imaging of heart (**A**) and blood (**C**) 24 h later and quantification of total radiant efficiency of heart (**B**) and blood (**D**). n ≥ 3 mice/group. Data are represented as mean ± SD.


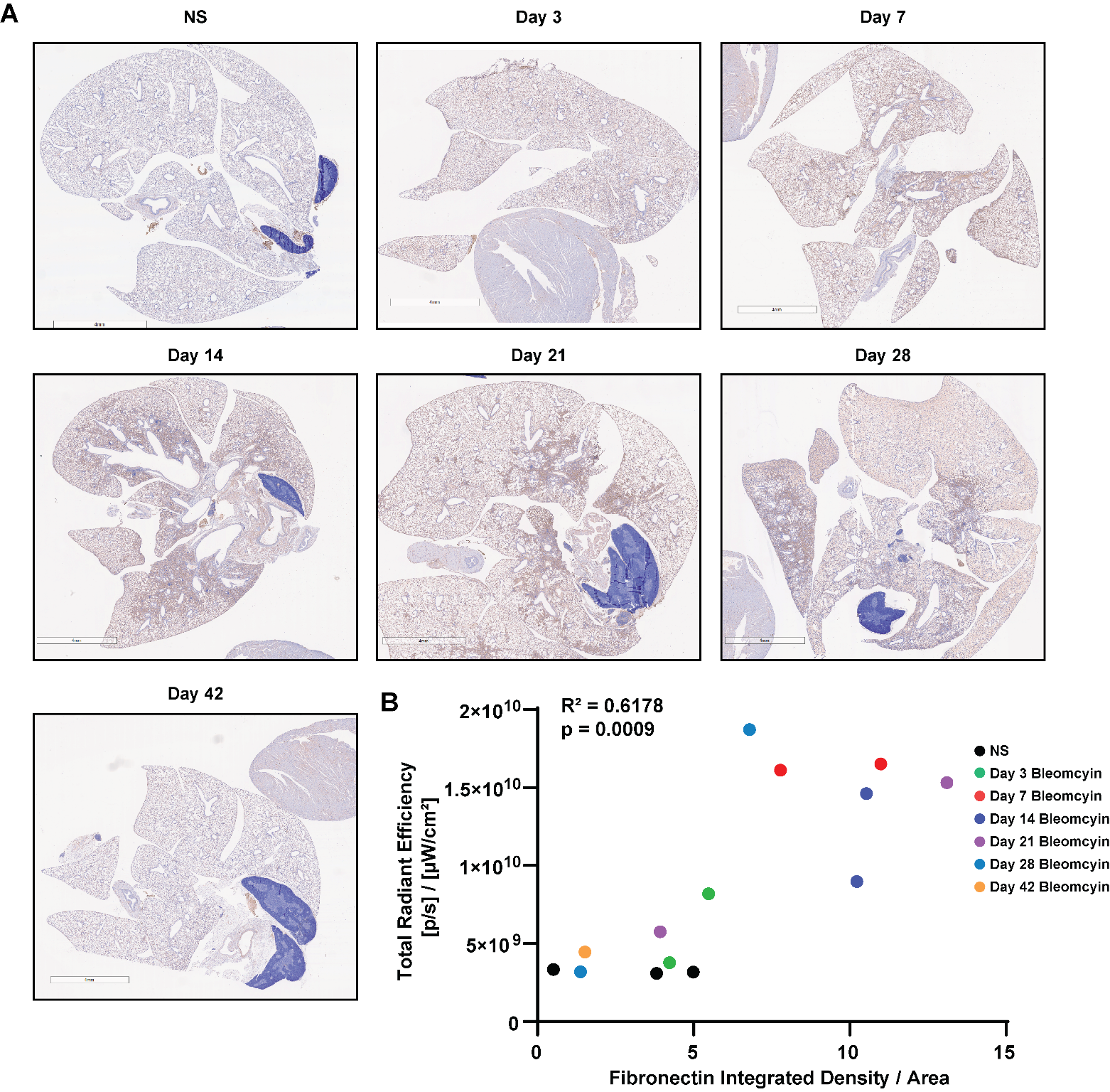


**Figure S4: Fibronectin expression correlates with Cy5-PEG-FUD uptake during the bleomycin-induced model of pulmonary fibrosis.** Mice were treated with bleomycin (1 U/kg, IT) or normal saline prior to the subcutaneous administration of Cy5-PEG-FUD in PEG-FUD (0.1875 mg/kg Cy5-PEG-FUD in 12.5 mg/kg mass dose). One day later, mouse lung tissues were imaged *ex vivo* using the In Vivo Imaging System and total radiant efficiency quantified. The same tissues were subsequently sectioned, subjected to immunohistochemical staining against fibronectin and subsequent signal quantification. **A.** Representative images are shown at indicated time points. **B.** The total radiant efficiency and fibronectin expression were subjected to linear regression modeling for indicated time points.

**Table 1 – Single dose toxicity blood analyses p-value list in the indicated comparisons**

| **Analyte** | **Dose (mg/ml)** | **PEG-FUD v. PEG-mFUD (p value)** | **PEG-FUD v. Vehicle (p value)** | **PEG-mFUD v. Vehicle (p value)** |
| --- | --- | --- | --- | --- |
| WBC (10^9 /L) | 1.6 | 0.573580273 | 0.175440104 | *0.048465426* |
|  | 3.2 | 0.507715481 | 0.143860913 | 0.18345002 |
|  | 6.4 | 0.735824115 | 0.530475288 | 0.836475788 |
|  | 12.5 | 0.521356531 | 0.079270164 | 0.340800613 |
|  | 25 | 0.280481636 | 0.287114631 | 0.067706375 |
| LYM (10^9 /L) | 1.6 | 0.812996338 | 0.081894191 | 0.054467461 |
|  | 3.2 | 0.937502436 | 0.24523887 | 0.237069539 |
|  | 6.4 | 0.625278539 | 0.661489119 | 0.834712529 |
|  | 12.5 | 0.609050557 | 0.063902077 | 0.238695906 |
|  | 25 | 0.494478737 | 0.064534503 | 0.067219399 |
| MON (10^9/L) | 1.6 | 0.610290175 | 0.862324778 | 0.719670799 |
|  | 3.2 | 0.212454541 | 0.629886441 | 0.454904224 |
|  | 6.4 | 0.930716811 | 0.87005784 | 0.93417385 |
|  | 12.5 | 0.623574199 | 0.570418201 | 0.871888771 |
|  | 25 | 0.320203291 | 0.459714695 | 0.863842318 |
| NEUT (10^9 /L) | 1.6 | *0.043642582* | 0.314200109 | 0.686311076 |
|  | 3.2 | 0.428846596 | 0.942899854 | 0.685781265 |
|  | 6.4 | *0.012435917* | 0.573154856 | 0.569030087 |
|  | 12.5 | 1 | 0.809854879 | 1 |
|  | 25 | 0.169707196 | 0.470393004 | 0.809197082 |
| LYM % | 1.6 | 0.125541126 | 0.329004329 | 1 |
|  | 3.2 | 0.599750653 | 0.488765865 | 0.399148557 |
|  | 6.4 | *0.000612786* | 0.808108781 | 0.148530316 |
|  | 12.5 | 0.818181818 | 0.937229437 | 1 |
|  | 25 | 0.052073103 | 0.382116011 | 0.501875364 |
| MON % | 1.6 | 0.500985815 | 0.898394644 | 0.380862155 |
|  | 3.2 | 0.1911972 | 0.799400656 | 0.345954397 |
|  | 6.4 | 0.936074677 | 1 | 1 |
|  | 12.5 | 0.868840056 | 0.473979192 | 0.644576588 |
|  | 25 | 0.320307214 | 0.35073944 | 0.88362022 |
| NEUT % | 1.6 | 0.081439732 | 0.313092754 | 0.935962473 |
|  | 3.2 | 0.445221445 | 0.830094265 | 0.935736281 |
|  | 6.4 | *0.015936571* | 0.469612624 | 0.572475574 |
|  | 12.5 | 0.936074677 | 0.747491101 | 0.935736281 |
|  | 25 | 0.108694581 | 0.377642391 | 0.57450381 |
| RBC (10^12 /L) | 1.6 | 0.269757864 | 0.252921041 | 0.953869367 |
|  | 3.2 | 0.769162042 | 0.474343753 | 0.463260214 |
|  | 6.4 | 0.470393004 | 0.24025974 | 0.228950681 |
|  | 12.5 | 0.688028379 | 0.826028153 | 0.952339195 |
|  | 25 | 1 | 0.818181818 | 1 |

| **Analyte** | **Dose (mg/ml)** | **PEG-FUD v. PEG-mFUD (p value)** | **PEG-FUD v. Vehicle (p value)** | **PEG-mFUD v. Vehicle (p value)** |
| --- | --- | --- | --- | --- |
| HGB (g/dL) | 1.6 | 0.866920092 | 0.308106111 | 0.446579199 |
|  | 3.2 | 0.758264269 | 0.489777211 | 0.431948263 |
|  | 6.4 | *0.039885608* | 0.523086694 | *0.036252742* |
|  | 12.5 | 0.877413087 | 0.934914705 | 0.836934486 |
|  | 25 | 0.119173025 | 0.939665295 | 0.136010274 |
| HCT % | 1.6 | 0.235324764 | 0.116300217 | 0.943375121 |
|  | 3.2 | 0.765781266 | 0.997602393 | 0.524936405 |
|  | 6.4 | 0.052380929 | 0.089356261 | 0.665535766 |
|  | 12.5 | 0.913755085 | *0.006073139* | *0.003321622* |
|  | 25 | 0.191324962 | *0.0110062* | *0.000366336* |
| MCV (fl) | 1.6 | 0.841338192 | 0.64111725 | 0.49242727 |
|  | 3.2 | 0.510879141 | 0.445460487 | 0.437185523 |
|  | 6.4 | 0.240606493 | *0.003600915* | *0.00634496* |
|  | 12.5 | 0.306056079 | *0.005083806* | *0.00634496* |
|  | 25 | 0.232430482 | *0.00777632* | *0.003793776* |
| MCH (pg) | 1.6 | 0.082699963 | 0.875805588 | 0.159870651 |
|  | 3.2 | 0.885812469 | 0.849472897 | 0.947976117 |
|  | 6.4 | 0.176791527 | 0.128101701 | 0.819323359 |
|  | 12.5 | 0.846560703 | 0.556284383 | 0.729755886 |
|  | 25 | *0.032796467* | 0.935736281 | 0.14164469 |
| MCHC (g/dL) | 1.6 | 0.173275815 | 0.592780701 | 0.311815193 |
|  | 3.2 | 0.159053523 | 0.102254878 | 0.681871938 |
|  | 6.4 | 0.304891386 | *0.018339491* | *0.007534865* |
|  | 12.5 | 0.676147512 | *0.001742692* | *0.003580059* |
|  | 25 | 0.492495616 | *0.001044583* | *0.002017161* |
| RDWc % | 1.6 | 1 | *0.027271591* | *0.008980671* |
|  | 3.2 | 1 | *0.003146912* | *0.004624229* |
|  | 6.4 | 0.098090519 | *0.004479259* | *0.004624229* |
|  | 12.5 | 0.56406414 | *0.004697697* | *0.004624229* |
|  | 25 | 1 | *0.004624229* | *0.004624229* |
| RDWs fl | 1.6 | 0.92410231 | 0.156039402 | 0.114710478 |
|  | 3.2 | 0.102734594 | *0.003105035* | *0.003537936* |
|  | 6.4 | 0.195346301 | *0.004551416* | *0.004551416* |
|  | 12.5 | 0.301210795 | *0.004624229* | *0.004551416* |
|  | 25 | 0.501726757 | *0.004771822* | *0.004551416* |
| PLT (10^9 /L) | 1.6 | 0.647328685 | 0.930735931 | 0.688403863 |
|  | 3.2 | 0.533799534 | 0.365967366 | 0.588744589 |
|  | 6.4 | 0.818181818 | 0.30952381 | 0.484848485 |
|  | 12.5 | 1 | 0.688403863 | 0.588744589 |
|  | 25 | 0.484848485 | 0.699134199 | 0.937229437 |

| **Analyte** | **Dose (mg/ml)** | **PEG-FUD v. PEG-mFUD (p value)** | **PEG-FUD v. Vehicle (p value)** | **PEG-mFUD v. Vehicle (p value)** |
| --- | --- | --- | --- | --- |
| PCT % | 1.6 | 0.645055235 | 0.27113235 | *0.043452802* |
|  | 3.2 | 1 | 0.09947808 | 0.092125104 |
|  | 6.4 | 0.935736281 | *0.012435917* | 0.064610089 |
|  | 12.5 | 0.935622282 | 0.571793054 | 0.629149731 |
|  | 25 | 0.469612624 | 0.747920928 | 0.148115796 |
| MPV fl | 1.6 | 0.351266014 | 0.141384415 | *0.019158923* |
|  | 3.2 | 0.827501027 | 0.070970367 | 0.052135088 |
|  | 6.4 | 0.619714773 | *0.01212662* | 0.089840769 |
|  | 12.5 | 1 | 0.257280177 | 0.294551629 |
|  | 25 | 0.367359658 | 0.743971478 | 0.188173875 |
| PDWc % | 1.6 | 0.338351722 | 0.641954583 | 0.93333408 |
|  | 3.2 | 1 | 0.610120155 | 0.51286177 |
|  | 6.4 | 0.387981916 | 0.391804855 | 0.252134911 |
|  | 12.5 | 1 | 0.514392875 | 0.464053071 |
|  | 25 | 0.934326882 | 0.418415302 | 0.626702406 |
| PDWs fl | 1.6 | 0.338351722 | 1 | 0.498403924 |
|  | 3.2 | 0.73381137 | 0.106964985 | 0.17800989 |
|  | 6.4 | 0.314426562 | 0.089800436 | *0.047761271* |
|  | 12.5 | 1 | 0.851762062 | 0.779752998 |
|  | 25 | 0.934326882 | 0.711209719 | 1 |
| BUN (mg/dL) | 1.6 | 0.373298061 | 0.119982392 | 0.409653723 |
|  | 3.2 | *0.044941084* | *0.002767784* | *0.020445895* |
|  | 6.4 | 0.261672114 | *0.004075476* | *0.00313182* |
|  | 12.5 | 0.159402657 | *0.001027273* | *0.025581541* |
|  | 25 | *0.026071542* | *0.000104051* | *0.000785095* |
| CRE (mg/dL) | 1.6 | 0.64111725 | 0.833028894 | 0.441418327 |
|  | 3.2 | 0.404656762 | 0.070532849 | 0.282425348 |
|  | 6.4 | 0.404656762 | 0.070532849 | 0.282425348 |
|  | 12.5 | 0.67328998 | 0.441418327 | 0.855132141 |
|  | 25 | 0.404656762 | 0.282425348 | 0.070532849 |
| ALT (U/L) | 1.6 | 0.247264086 | 0.908686577 | 0.194199224 |
|  | 3.2 | 0.145968903 | 0.687362716 | 0.685781265 |
|  | 6.4 | 0.259817508 | 0.570418201 | 1 |
|  | 12.5 | *0.023470611* | 0.073486725 | 1 |
|  | 25 | *0.022000543* | 0.571107272 | *0.004697697* |
| ALP (U/L) | 1.6 | 0.091658746 | 0.29704927 | 0.62390808 |
|  | 3.2 | 0.271970246 | 0.698776619 | 0.650172662 |
|  | 6.4 | 0.576366334 | 0.551989252 | 0.336830352 |
|  | 12.5 | 0.687884591 | 0.630355526 | 0.818181818 |
|  | 25 | 1 | 0.093073593 | *0.037040731* |

| **Analyte** | **Dose (mg/ml)** | **PEG-FUD v. PEG-mFUD (p value)** | **PEG-FUD v. Vehicle (p value)** | **PEG-mFUD v. Vehicle (p value)** |
| --- | --- | --- | --- | --- |
| AST (U/L) | 1.6 | 0.272229256 | 0.583024912 | 0.809526832 |
|  | 3.2 | 0.630355526 | 0.393939394 | 0.937229437 |
|  | 6.4 | 0.171978682 | 0.171978682 | 0.872559031 |
|  | 12.5 | 0.872559031 | 0.630355526 | 0.588744589 |
|  | 25 | 0.393939394 | 0.699134199 | 0.699134199 |
| TBIL (mg/dL) | 1.6 | 0.098960154 | 0.220671362 | 0.640373539 |
|  | 3.2 | 0.112195824 | 0.594792522 | 0.31086287 |
|  | 6.4 | *0.034094293* | 0.112195824 | 0.594792522 |
|  | 12.5 | 0.173945017 | 0.173945017 | 1 |
|  | 25 | 0.404656762 | 0.594792522 | 0.173945017 |
| GLU (mg/dL) | 1.6 | 0.768410605 | 0.422455401 | 0.449032958 |
|  | 3.2 | 0.588525825 | 0.213156621 | 0.070143004 |
|  | 6.4 | 0.118264081 | 0.983982393 | 0.203594931 |
|  | 12.5 | 0.277617795 | 0.50604446 | 0.970519881 |
|  | 25 | 0.924023099 | 0.537018006 | 0.477141837 |
| CA (mg/dL) | 1.6 | 0.871755287 | 0.984393397 | 0.954233767 |
|  | 3.2 | 0.340893132 | 0.505139171 | 0.265039412 |
|  | 6.4 | 0.42089282 | 0.172732829 | 0.260658124 |
|  | 12.5 | 0.42550337 | 0.191249025 | 0.530182646 |
|  | 25 | 0.487675033 | 0.385953632 | 0.225926137 |
| TP (g/dL) | 1.6 | 0.987160718 | 0.422141519 | 0.378944998 |
|  | 3.2 | 0.611835085 | 0.110027901 | *0.047666723* |
|  | 6.4 | 0.856613073 | 0.184832026 | 0.237908657 |
|  | 12.5 | 0.339800074 | 0.086618386 | 0.609453436 |
|  | 25 | 0.413811468 | *0.001365425* | *0.006541001* |
| ALB (g/dL) | 1.6 | 0.502039243 | 0.23091454 | 0.549014439 |
|  | 3.2 | 0.638622259 | 0.056485893 | *0.033483689* |
|  | 6.4 | 0.510127357 | 0.062586345 | 0.130804828 |
|  | 12.5 | 0.114818839 | *0.020376469* | 0.601187216 |
|  | 25 | 0.457200798 | *0.003642828* | *0.014671995* |
| GLOB (g/dL) | 1.6 | 0.321369611 | 0.617703171 | 0.393203382 |
|  | 3.2 | 0.791780773 | 0.700933577 | 1 |
|  | 6.4 | 0.51695351 | 0.330421984 | 0.866864531 |
|  | 12.5 | 0.476178589 | 0.195651156 | 0.856396245 |
|  | 25 | 0.743320547 | 0.202482194 | 0.525600654 |
| Na+ (mmol/L) | 1.6 | 1 | 0.557673806 | 0.490465555 |
|  | 3.2 | 0.548876718 | 0.933836067 | 0.809526832 |
|  | 6.4 | 0.836023264 | 0.41276357 | 0.457665125 |
|  | 12.5 | 0.760809188 | 0.286706646 | 0.502255611 |
|  | 25 | 0.6205149 | 0.849654046 | 0.581418885 |

| **Analyte** | **Dose (mg/ml)** | **PEG-FUD v. PEG-mFUD (p value)** | **PEG-FUD v. Vehicle (p value)** | **PEG-mFUD v. Vehicle (p value)** |
| --- | --- | --- | --- | --- |
| K+ (mmol/L) | 1.6 | 0.23319714 | 0.169929895 | 0.871662991 |
|  | 3.2 | 0.871436012 | 0.091554241 | 0.064610089 |
|  | 6.4 | 0.054241118 | 0.872336781 | 0.127537709 |
|  | 12.5 | 0.818181818 | 0.297106984 | 0.148115796 |
|  | 25 | 1 | 0.092125104 | *0.044951243* |
| Cl- (mmol/L) | 1.6 | *0.042200819* | 0.115606437 | 0.566922585 |
|  | 3.2 | 0.871207824 | 0.566922585 | 0.685781265 |
|  | 6.4 | 0.685781265 | 0.870747781 | 0.871888771 |
|  | 12.5 | 0.323642454 | *0.008856284* | 0.102470435 |
|  | 25 | 0.870048378 | 0.465659066 | 0.516662941 |
| tCO2 (mmol/L) | 1.6 | 0.766760432 | 0.404228368 | 0.266289927 |
|  | 3.2 | 0.914148769 | 0.22737668 | 0.240051636 |
|  | 6.4 | 0.938431688 | 0.370954987 | 0.347079689 |
|  | 12.5 | 0.93333408 | 1 | 0.936074677 |
|  | 25 | 1 | 0.871662991 | 0.747920928 |
